# Comparative transcriptomic analysis of immune responses of the migratory locust, *Locusta migratoria*, to challenge by the fungal insect pathogen, *Metarhizium acridum*

**DOI:** 10.1186/s12864-015-2089-9

**Published:** 2015-10-26

**Authors:** Wei Zhang, Jianhong Chen, Nemat O. Keyhani, Zhengyi Zhang, Sai Li, Yuxian Xia

**Affiliations:** Genetic Engineering Research Center, School of Life Sciences, Chongqing University, Chongqing, 400045 People’s Republic of China; Department of Microbiology and Cell Science, Institute of Food and Agricultural Sciences, University of Florida, Gainesville, FL 32611 USA; Chongqing Engineering Research Center for Fungal Insecticide, Chongqing, 400045 People’s Republic of China; Key Laboratory of Gene Function and Regulation Technologies under Chongqing Municipal Education Commission, Chongqing, 400045 People’s Republic of China

**Keywords:** Locust, *Locusta migratoria*, Insect immunity, Fat body, Hemocyte, Entomopathogen, Fungal virulence, *Metarhizium acridum*

## Abstract

**Background:**

The migratory locust, *Locusta migratoria manilensis,* is an immensely destructive agricultural pest that forms a devastating and voracious gregarious phase. The fungal insect pathogen, *Metarhizium acridum*, is a specialized locust pathogen that has been used as a potent mycoinsecticide for locust control. Little, however, is known about locust immune tissue, i.e. fat body and hemocyte, responses to challenge by this fungus.

**Methods:**

RNA-seq (RNA sequencing) technology were applied to comparatively examine the different roles of locust fat body and hemocytes, the two major contributors to the insect immune response, in defense against M. acridum. According to the sequence identity to homologies of other species explored immune response genes, immune related unigenes were screened in all transcriptome wide range from locust and the differential expressed genes were identified in these two tissues, respectively.

**Results:**

Analysis of differentially expressed locust genes revealed 4660 and 138 up-regulated, and 1647 and 23 down-regulated transcripts in the fat body and hemocytes, respectively after inoculation with *M. acridum* spores. GO (Gene Ontology) enrichment analysis showed membrane biogenesis related proteins and effector proteins significantly differentially expressed in hemocytes, while the expression of energy metabolism and development related transcripts were enriched in the fat body after fungal infection. A total of 470 immune related unigenes were identified, including members of the three major insect immune pathways, i.e. Toll, Imd (immune deficiency) and JAK/STAT (janus kinase/signal transduction and activator of transcription). Of these, 58 and three were differentially expressed in the insect fat body or hemocytes after infection, respectively. Of differential expressed transcripts post challenge, 43 were found in both the fat body and hemocytes, including the *Lm*Lys4 lysozyme, representing a microbial cell wall targeting enzyme.

**Conclusions:**

These data indicate that locust fat body and hemocytes adopt different strategies in response to *M. acridum* infection. Fat body gene expression after *M. acridum* challenge appears to function mainly through activation of innate immune related genes, energy metabolism and development related genes. Hemocyte responses attempt to limit fungal infection primarily through regulation of membrane related genes and activation of cellular immune responses and release of humoral immune factors.

**Electronic supplementary material:**

The online version of this article (doi:10.1186/s12864-015-2089-9) contains supplementary material, which is available to authorized users.

## Background

The migratory locust, *Locusta migratoria manilensis*, undergoes a striking behavioral phase transition, from a solitary, essentially harmless animal to a seemingly endless cloud of voracious, traveling swarms that devour the vegetation in their path. Both significant historical and modern accounts of their rampaging effects have been recorded. Continued outbreaks and infestations in Madagascar, the Red Sea coast, and in the Caucasus and Central Asia are but several recent examples [1]. Invertebrates, including locusts, lack the ability to produce true antibodies as part of adaptive immune responses, and instead rely on innate mechanisms for mitigating microbial infections, consisting of cellular and humoral responses [2]. Humoral factors include the production of oxygen and nitrogen free radicals, anti-microbial peptides (AMPs), and enzyme cascades, e.g. the prophenoloxidase (PPO) pathway, the latter of which produce cytotoxic quinones and mediate coagulation and melanization responses upon pathogen detection [3]. Many humoral compounds are produced and secreted into the hemocoel by the fat body, the main insect organ of intermediate metabolism [3]. The fat body produces most of the proteins and metabolites found in the hemolymph, and acts as the central controller of the synthesis and utilization of energy reserves, i.e. glycogen and lipids [4]. This organ also functions to sequester, and release upon appropriate (hormonal) signal, proteins or other molecules required for morphogenesis, egg maturation, and lipid/hormone transport, examples of which include growth factors, vitellogenins, and lipophorins, respectively [5].

Insects also have macrophage-equivalent cells termed hemocytes that circulate in the hemolymph and are capable of secreting a number of humoral immune factors. One of the main activities of hemocytes is to phagocytose foreign cells and material. Hemocyte aggregation seeks to isolate and entrap foreign materials within the hemocoel, resulting in nodule formation and encapsulation that is coupled with melanization via PPO activation, thus linking aspects of the humoral and cellular responses [6]. Insect hemocytes are produced by stem cells that are mesodermally located, and differentiate into a variety of different morphologically and functionally distinct lineages. Depending upon the insect, a combination of a number of the most common types of hemocytes, i.e. granulocytes, prohemocytes, plasmatocytes, spherulocytes, and/or oenocytoids are produced [7]. One mechanism of hemocytes action occurs via recognition of foreign microbial targets by pattern recognition receptors (PPRs) that respond to pathogen-associated molecular pattern (PAMP) molecules found on microorganisms. Significant information concerning insect immune systems is known for dipterans, i.e. the *Drosophila* model system as well as various mosquito species [8, 9], hymenopteran honey bees, *Apis mellifera* [10], and more recently the coleopteran (beetles) insect *Tribolium castaneum* [11]. A variety of immune related genes have been shown to be expressed in insect hemocytes and fat bodies. In the mosquito, *Anopheles gambia*, 1053 genes were found to be predominantly expressed in adult female hemocytes [12], with 13 and 44 immune related genes were differentially transcribed in *A. gambiae* hemocytes after challenged by *Escherichia coli* or *Micrococcus luteus* [13]. In the mosquito species, *Aedes aegypti* and *Armigeres subalbatus*, 169 and 103 immune related EST clusters were identified in hemocytes, with a small subset, 11 and 16 genes, differentially expressed after bacterial inoculation, respectively [14]. In the tsetse fly, *Glossinia morsitans*, 60 putative immunity-pathway-related genes were identified in the fat body [15], and 80 putative immune clusters were identified in the fat body of *Antheraea mylitta* after challenged by *Escherichia coli* [16]. Whole body transcriptional responses of several insects, including of the corn borer, *Ostrinia furnacalis* and the whitefly, *Bemesia tabaci* to infection by *B. bassiana* have been reported [17, 18]. Although changes in the expression of various immune-related genes were noted, such whole organism approaches are likely to obscure significant portions of the immune response, and information concerning the reactions of specific immune-related tissues is lacking. In addition, far less is known concerning both general and specific microbial immune responses of hemimetabolous orthopterans that include locusts, than their holometabolous counterparts.

Insect pathogenic fungi, especially *Beauveria* and *Metarhizium* species, have long been considered as potential pest biological control agents, with several commercial products currently available worldwide [19, 20]. Entomogenous fungi have been suggested to engage in a co-evolutionary arms race with their insect hosts [21]. These agents pose less environmental risk than chemical insecticides and are compatible with organic and sustainable farming practices, notably available in developing countries. While many entomopathogenic fungi are broad host range insect pathogens, e.g. *B. bassiana* and *M. roberstsii*, several lineages have evolved high but restricted virulence towards certain insect hosts [22, 23]. *M. acridum* is one such restricted host-range species, particularly effective against orthopteran insects and has been used in both Africa and Asia as an effective agent as part of Integrated Pest Management (IPM) programs for locust control [24]. Infection occurs via attachment of fungal spores (conidia) to host surfaces, followed by germination and penetration of the insect cuticle [25]. As penetrating hyphae reach the hemocoel, they produce single celled structures that are capable of evading various immune reactions including hemocytes and fat body activated antimicrobial responses [26, 27]. Death of the host typically occurs within 3–7 days, after which the fungus sporulates on the dead insect, producing cells capable of infecting another round of hosts. A number of genes implicated in *M. acridum* virulence have been characterized, and intriguingly, addition of a single esterase gene can expand the host range of *M. acridum* to certain Leptidoperans [28–30].

Here, we explored the transcriptional responses of critical host immune tissues, namely the hemocytes and the fat body, of *L. migratoria*, to fungal infection by *M. acridum*. Transcriptomic analysis revealed that hemocytes and the fat body adopt distinct strategies in response to infection by the fungus. A relatively small number of genes were affected in hemocytes, which appeared to elevate the expression of pathways for the release of reactive nitrogen intermediates (RNI), and alter aspects of membrane, potentially to facilitate phagocytosis or engulfment pathways involved in defense against fungi. In contrast, a larger set of genes was activated in the fat body including those affecting known classical antimicrobial pathways, e.g. Toll and JAK/STAT. In addition, altered expression of cell differentiation and maturation genes, and changes in basic energy metabolism were noted, perhaps linked to an increase in metabolic energy needed in attempts to thwart the infection. Analysis of conserved immune pathways led to their identification in *L. migratoria* (~470 transcripts). Among them, 59 (13 %) and three (6.5 %) showed significant differential expression in the fat body and hemocytes, respectively, after *M. acridum* infection. These results reveal the transcriptional responses of the fat body and hemocytes in defense against a locust specific entomopathogenic fungus, and detail immune related genes in the transcriptome of the Orthopteran, *L. migratoria manilensis*.

## Methods

### Insects, fungal strains, and inoculation protocol

Adult males of the migratory locust, *Locusta migratoria manilensis* (Orthoptera: Acrididae), reared until 5 days after final ecdysis were used in all experiments. Locusts were maintained in metal cages at 30 ± 3 °C with 70–75 % relative humidity and a photoperiod of 16 h light, 8 h dark, and supplied with fresh wheat shoots, wheat bran (supplemented with dried brewer’s yeast) and water as described [31]. The fungal strain *M. acridum* CQMa102, was isolated from the yellow-spined bamboo locust, *Ceracris kiangsu* Tsai, by the Genetic Engineering Center of Chongqing University. The fungus was grown on one-quarter strength Sabouraud dextrose agar (SDA) for 15 days at 28 °C, after which conidia were harvested and suspended in cottonseed oil. Mycelia were removed by filtration through sterile lens paper. The concentration of spores was determined using a Neubauer haemocytometer. Healthy locusts were selected and inoculated with 5 μl of conidial suspensions adjusted to 1 × 10^8^ conidia as described [32]. Control insects were treated with the same volume of cottonseed oil. Treated insects were reared in individual cages. Assay were performed using 20 locusts and each assay was repeated three times.

### Sample preparation, library construction and sequencing

Locusts were collected after 8, 16, 24, and 32 h post infection, respectively. For each time course, ~30 infected and 30 control male adults were placed on ice to anesthetize them and the fat bodies were dissected in a Petri dish on ice in locust physiological saline solution (LoPS, 150 mM NaCl, 10 mM KCl, 4 mM CaCl_2_, 2 mM MgCl_2_, 4 mM NaHCO_3_, 5 mM 4-(2-hydroxyethyl)-1-piperazine ethanesulphonic acid pH 7.2, 0.1 % Ficoll). Hemolymph were collected from locusts at the same time as described [33]. The dissected fat bodies and hemocyte were immediately transferred to mortars containing liquid nitrogen and homogenized, followed by RNA isolation using the Trizol Reagent (Invitrogen) according to the manufacturer’s instructions. The RNA samples were further digested with ten units of DNase I (Takara, China) for 1 h at 37 °C to remove residual genomic DNA. The quantity and quality of the RNA samples were examined using a Nanodrop ND-1000 spectrophotometer (LabTech, USA) and an Agilent 2100 Bioanalyzer (Agilent Technologies, California, USA). Select RNA samples from different time courses derived from the same treatment and tissue were pooled in equal proportions to construct cDNA libraries using TruSeq RNA Sample Prep Kit v2 (Illumina, USA) following manufacturer’s instructions. Briefly, poly(A) mRNA from 1 μg of total RNA was isolated using oligo(dT)-conjugated magnetic beads. Purified mRNAs were fragmented (200 nt to 700 nt) and reverse transcribed into cDNA using Super Script II Reverse Transcriptase (Invitrogen, USA). Second-strand cDNA was synthesized in reaction mixtures containing 1× buffer, dNTPs, RNase H, and DNA polymerase I. Short fragments were purified using the Agencourt® AMPure® XP beads (Beckman Coulter Inc., Beverly, MA, USA) and resolved with EB buffer for end repair and single nucleotide A (adenine) tailing. Fragments were then connected with sequencing adapters, and enriched by 15 cycles of PCR amplification to obtain adequate fragments for the final cDNA library. The quantification and qualification of the cDNA library were assessed by an Agilent 2100 Bioanalyzer and ABI Step One Plus Real-Time PCR System, and sequenced on the Illumina HiSeq™ 2000 pair-end system (Illumina, USA). Illumina sequencing was performed at the Beijing Genomics Institute (BGI-Shenzhen, China).

### Assembly and annotation of transcriptomes

All raw transcriptome data generated from Illumina sequencing were deposited in the SRA database (NCBI) with accession number SRX1036497 (fat body control), SRX1036507 (fat body infected), SRX1036511 (hemocyte control) and SRX1036517 (hemocyte infected). Before assembling the clean reads, the raw reads were preprocessed using filter-fq software. The raw reads containing only adaptor, sequences, reads with >5 % unknown nucleotides, and low quality reads (reads containing more than 20 % bases with Q-value ≤10) were removed. The obtained clean read datasets were assembled using Trinity (release 20130225) [34] under default parameters, except for min_kmer_cov and min_glue set at three. The four SRA datasets in our experiment were assembled separately at the beginning. Another 37 locust SRA datasets were downloaded from the NCBI. Among these downloaded SRA datasets, those who have same sequencing lengths were assembled separately similarly. These 41 partially assembled datasets then were combined to assemble together according to the following processes. The Trinity-based assembled unigenes from each sample were further processed using the Clustering software TGICL platform [35], to identify sequence splicing and redundancy, resulting in data containing the non-redundant unigenes of maximum sequence length. These resultant unigene datasets from each sample were then assembled into a unique “All-unigene sequence” dataset using the TGICL platform. The All-unigene sequences were then separated into two classes: clusters (CL, collection of homologous unigenes, i.e. sharing >70 % sequence identity) and singletons (unigene).

The unigene sequence datasets were annotated using available protein databases including Nr (non-redundant protein databases), SwissProt, KEGG (Kyoto Encyclopedia of Genes and Genomes) and COG (Cluster of Orthologous Groups), using the blastx algorithm (http://www.ncbi.nlm.nih.gov/) with a cut-off E-value of 10^−5^ [36]. Provisional protein functional information was assigned from comparative annotation to the most similar protein in those databases.

### Differentially expression and immune related unigene analysis

Fat body and hemocytes genes that were differentially expressed between control and infected populations were identified, respectively, using a table of counts constructed with fragments per kb per million fragments (FPKM) values, which adjusted the number of fragments by the total number of fragments mapped and the length of the gene [37, 38]. Difference gene expression (DEG) of fat body and hemcoyte between control and infection group were determined by using FPKM value under the standard of false discovery rate (FDR) <0.001 and an absolute value of the log_2_ratio >1.

The locust transcriptome database was used as the background to search for GO terms enriched within the DEG dataset using the hypergeometric test and a *p*-value <0.05 as the parameters for determining significantly enriched terms. Similarly, pathways significantly enriched with the DEG datasets were identified by mapping all differentially expressed genes to terms in the KEGG database using the hypergeometric test with a *p*-value <0.05.

Immune related genes were preliminary identified via screening using the BLASTX search algorithm against immune-related family members downloaded from the orthodb database (http://cegg.unige.ch/orthodb7), which included Insecta, waterflea and tick sequences. Searches were parameterized with a cut-off E-value of <10^−5^. Putative immunity-related genes were further analyzed by comparing their protein domains with the deduced protein domains of different family members. Protein domains are determined using Pfam (http://www.sanger.ac.uk/Software/Pfam/) and SMART (http://smart.embl.de/). Manual screening was further performed to verify all identified immune related genes, which were classified into different immune-gene related families.

### Quantitative RT-PCR analyses

To validate the results of the DEG, the expression change between control and fungal infected groups were examined by qRT-PCR for all of immune-related differentially expressed genes in the fat body (58 total) and hemocyte (three total) transcriptomes. Specific primers were designed for each gene and are listed in Additional file 1: Table S1. Total RNA from each sample was extracted as described above. 1 μg total RNA was reverse-transcribed in a 20 μl reaction using the Primescript TM RT reagent kit (TaKaRa, China). qRT-PCR was conducted using the CFX96TM Real-Time System (Bio-Rad, Hercules, CA, USA) with SYBR green (TaKaRa, China) using the following cycling parameters: 95 °C for 3 min, and 40 cycles of 95 °C for 5 s, 60 °C for 15 s, followed by melting curve generation from 65 to 95 °C. Primers designed to the *actin* genes were used as a reference control, and nuclease-free water was used as a negative control. All protocols for qRT-PCR experiments are in accordance with the Minimum Information Required for Publication of Quantitative Real-Time PCR Experiments guidelines [39]. Ct value was calculated from the results of three biological replications. The relative expression levels of each gene was analyzed according to 2^-ΔΔCT^ [ΔΔCt = ΔCt(test) - ΔCt (calibrator)] method [40].

## Results

### Illumina sequencing and read assembly

Locusts infected with spores of *M. acridum* and un-inoculated insects were used to generate cDNA libraries for examining insect responses to the fungal pathogen. The fat bodies and hemocytes of *M. acridum*-infected and untreated locusts were isolated and RNA purified from these tissues as detailed in the Methods section. Four cDNA libraries corresponding to control and fungal-treated, fat body and hemocyte derived tissues, respectively, were generated and sequenced using the Illumina platform. A total of 18.79 Gb of nucleotide sequences were obtained. Q20 percentages (sequencing error rate <1 %) and GC percentages for the different samples were as follows; (1) hemocyte (control), 95.56 and 40.31 %, (2) hemocyte treated, 94.19 and 40.23 %, (3) fat body (control), 93.89 and 41.55 %, and (4) fat body treated, 85.33 and 44.39 %, respectively. These GC percentages are in agreement with a previous report [41]. For assembly, 37 locust SRA datasets were downloaded from NCBI. After removal of adaptor sequences, ambiguous reads, and low quality reads (Q20 <20) from the combined (41 SRA) datasets, clean reads with same sequencing lengths were assembled separately, and then the resultant partial assembly further assembled together. In comparison with an analysis examining the separate assembly of our four datasets, the N50 and mean length of unigenes assembled from the combined SRAs dataset increased significantly, from 559–804 bp to 1607 bp and from 484–603 bp to 914 bp (Table 1), respectively. The total number of nucleotides (nt), mean nt length, N50 values, assembly into contigs, scaffolds, and the unigene sets for each condition/library is given in Table 1. These final mean lengths and N50 values are comparable or exceed that reported by others [42, 43]. Total assembled nucleotides was equal to 47.5G, representing a 22-fold coverage of the coding sequence space of the locust genome (~2.5G) [44], indicating a robust dataset and analysis pipeline for accurate sequence assembly and adequate transcriptome coverage. The final assembled sequences have been submitted to the NCBI Transcriptome Shotgun Assembly (TSA) Database under the accession #: GDIO00000000.

**Table 1:** Summary for the Illumina sequencing and de novo assembly of *Locusta migratoria manilensis* transcriptome

	Sample	Total number	Total length (nt)	Mean length (nt)	N50
Contig	FB Control	76,610	24,270,667	214	244
	FB Treated	90,480	35,948,413	248	302
	Hemo Control	102,738	37,214,667	241	298
	Hemo Treated	96,108	36,214,648	247	310
Scaffold	FB Control	78,784	25,901,123	329	442
	FB Treated	104,204	37,589,106	361	519
	Hemo Control	104,946	39,524,954	377	587
	Hemo Treated	98,818	38,331,782	388	621
Unigene	FB Control	44,235	21,396,869	484	559
	FB Treated	58,552	31,524,499	538	663
	Hemo Control	56,995	33,350,180	585	764
	Hemo Treated	53,979	32,541,902	603	804
	Together with SRA	50,809	46,450,242	914	1607

### Gene identification, functional annotation and classification

**Fig. 1:**
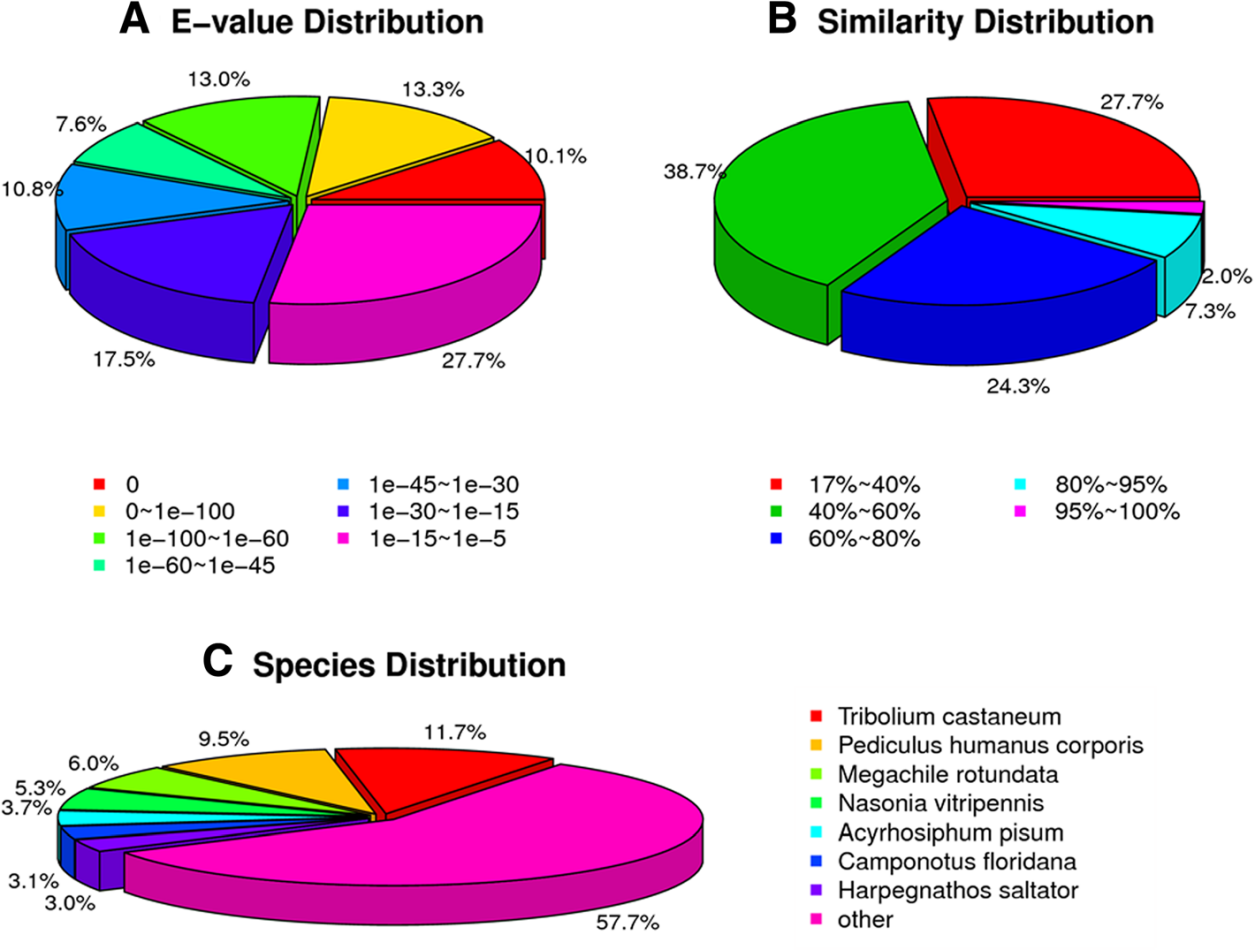
Summary of transcriptome analysis. **a** E-value distribution of BLAST hits for each unique sequence. **b** Similarity distribution of the top BLAST hits each sequence. **c** Species distribution shown as a percentage of the total homologous sequences with an E-value >e^−25^ using the first BLAST search hit for each sequence in the analysis

Among the 50,809 unigenes, approximately 22,698 were annotated. Within each cDNA dataset, 22,496, 19,009, 16,502 and 9781 unigenes could be provisionally annotated by searching against the Nr (non-redundant protein databases), SwissProt, KEGG (Kyoto Encyclopedia of Genes and Genomes) and COG (Cluster of Orthologous Groups), respectively. Searches performed using the Nr database resulted in the largest proportion of provisional annotations, producing hits for 44.3 % of all distinct sequences. Analysis of the E-value distribution for those sequences for which a hit could be identified in the Nr database, showed a relatively even distribution across the range parameters used, i.e. 10^−5^ >E-value >0 (Fig. 1a). Overall similarity for the majority of identified sequences (~70 %) was greater than 40 %, with the remainder (~30 %) showing similarity values ranging from 17 to 40 % (Fig. 1b). The species distribution for ~40 % of the best-matching sequences were seven other main insects, with the red flour beetle, *Tribolium castaneum* (11.7 %), and the human body louse, *Pediculus humanus corporis* (9.5 %), representing the top two insect species to which best-matching hits were seen (Fig. 1c). Approximately 58 % of the best-matching sequences were distributed to other organisms, e.g. *Apis mellifera* (2.4 %).

**Fig. 2:**
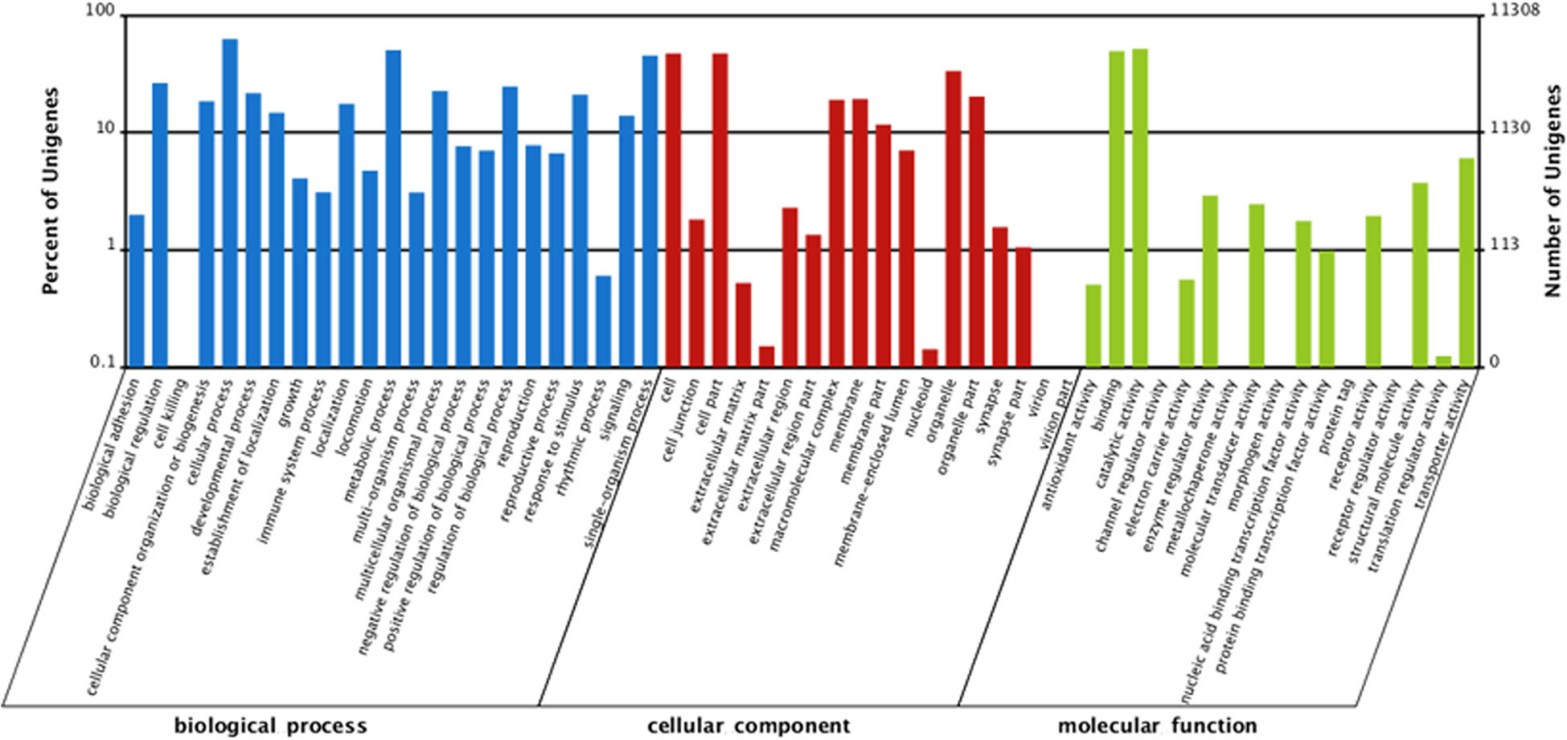
GO annotation of the overall unigene dataset. The total locust transcriptome dataset (9781 unigenes) were classified into biological process, cellular component, and molecular function subcategories

Unigene sequences were mapped using the international standardized gene functional classification (GO) system, which offers a dynamic-updated controlled vocabulary and a strictly defined concept to comprehensively describe properties of genes and their products in any organism. Annotated genes were binned into the three GO ontologies: molecular function, cellular component and biological process. Of the unigene set, 11,308 were annotated to at least one GO term. Among these, 8538 (75.5 %), 6281 (55.54 %), 9203 (81.38 %) unigenes were grouped into categories comprising biological process, cellular component and molecular function, respectively (Fig. 2). In the biological process, the most abundant subcategories were cellular processes (7064) and metabolic processes (5676). In the cellular component category, 348 unigenes were involved in immune system processes. The cell/cell part categories had 5308 unigenes assigned to them. Within the molecular function category, catalytic activity (enzymes, 5809) and binding (5565) were the two most abundant transcript annotation subcategories (Fig. 2).

**Fig. 3:**
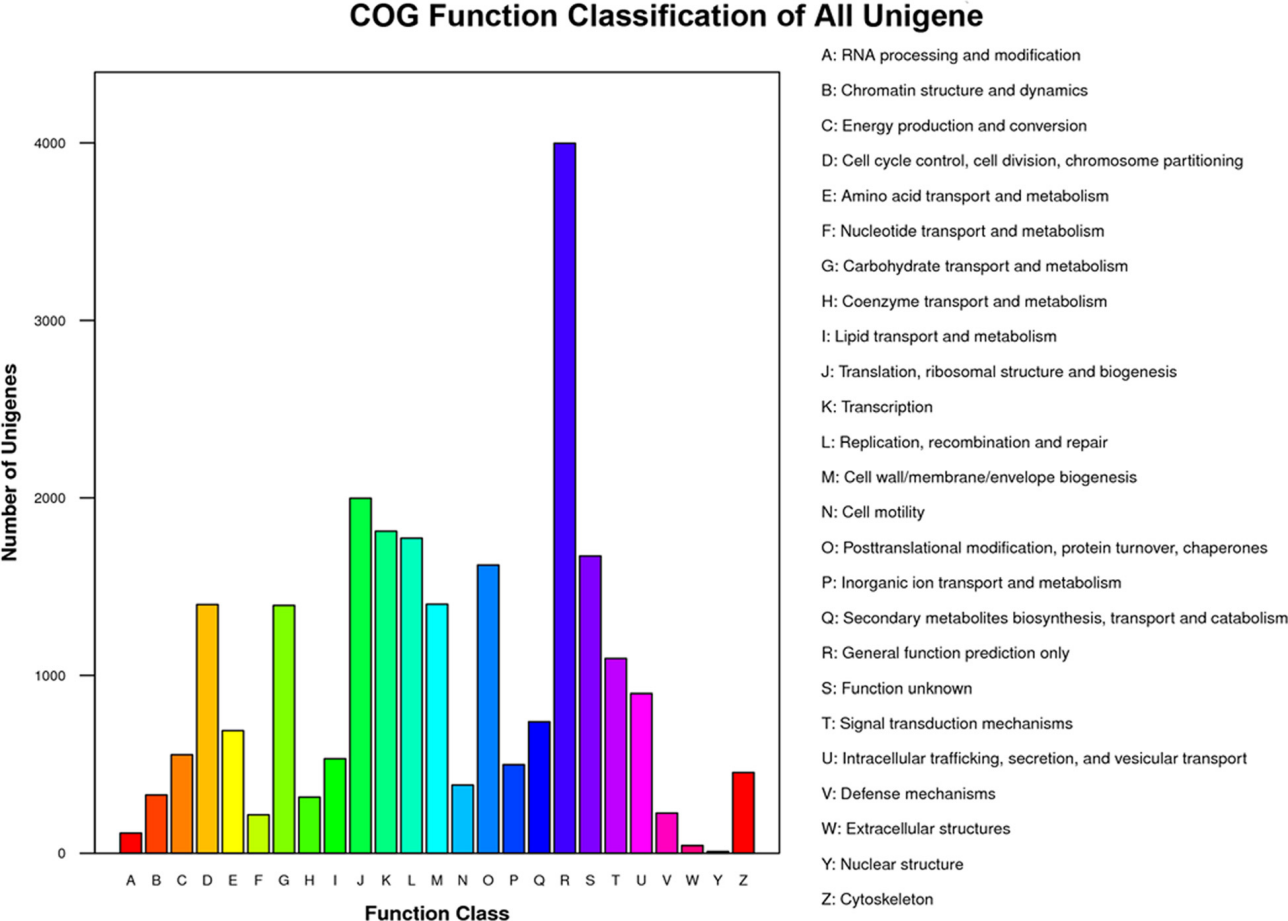
Analysis of clusters of orthologous groups of proteins (COGs). In all, 9781 unigenes were functionally grouped into the 25 COG categories and sub-catagories

COGs were delineated by comparing protein sequences encoded in complete genomes, representing major phylogenetic lineages. Each COG consists of individual proteins or groups of paralogs from at least 3 lineages and thus corresponds to an ancient conserved domain. In this study, 9781 unigenes were functionally grouped into the 25 COG categories. Of the total unigene set, 41 % identified within the COG annotation were found to belong to the subcategory of general function prediction (via the top hit). Translation, ribosomal structure and biogenesis were the second most abundant (1998 unigenes), followed by genes involved in transcription (1812 unigenes), replication, recombination and repair (1773 unigenes), and function unknown (1674). Among them, 2.3 % of unigenes were divided into the subcategory of defense mechanisms (Fig. 3).

### Identification of differentially expressed genes in response to *Metarhizium acridum* infection

Pairwise comparisons of the transcriptome of locust fat body and hemocyte tissues untreated and infected by *M. acridum* were performed. After infection by *M. acridum*, a large alteration in the transcriptome of the locust fat body was seen, with 4660 genes up-regulated and 1674 genes down-regulated. In contrast, the hemocyte response was much smaller with 161 and 23 genes up- and down-regulated, respectively, after infection by the fungus. The amplitude of the signals, i.e. the extent of transcriptional activation or repression, also differed between the fat body and hemocytes upon exposure to the pathogen. The fold changes (log_2_ ratio) of gene expression in the fat body ranged from −13.92 to 15.90, whereas in hemocytes the range was from −4.81 to 9.81.

**Fig. 4:**
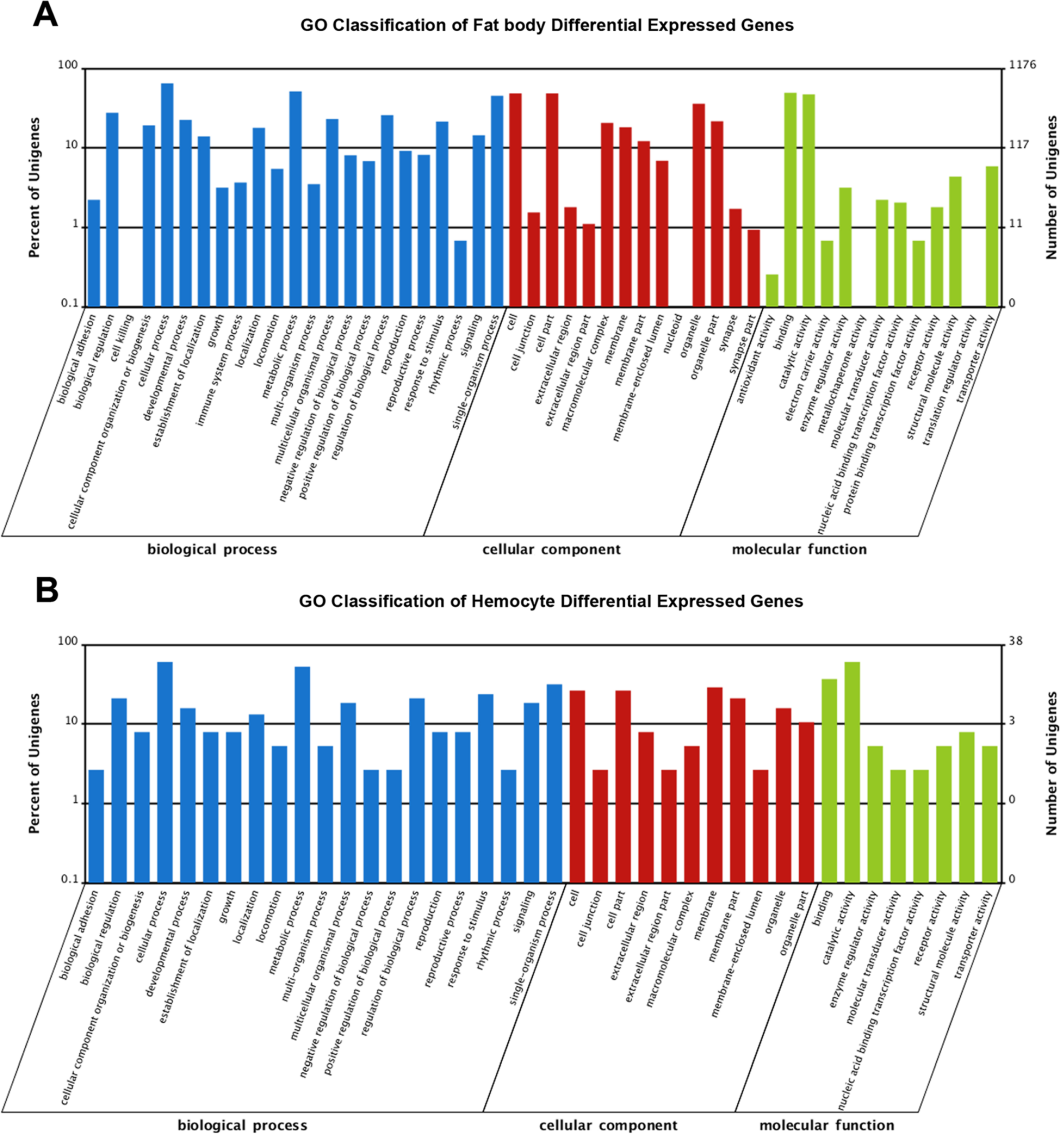
GO classification of differential expressed genes (DEGs) in the locust fat body (**a**) and in hemocytes (**b**) after *M. acridum* infection

**Fig. 5:**
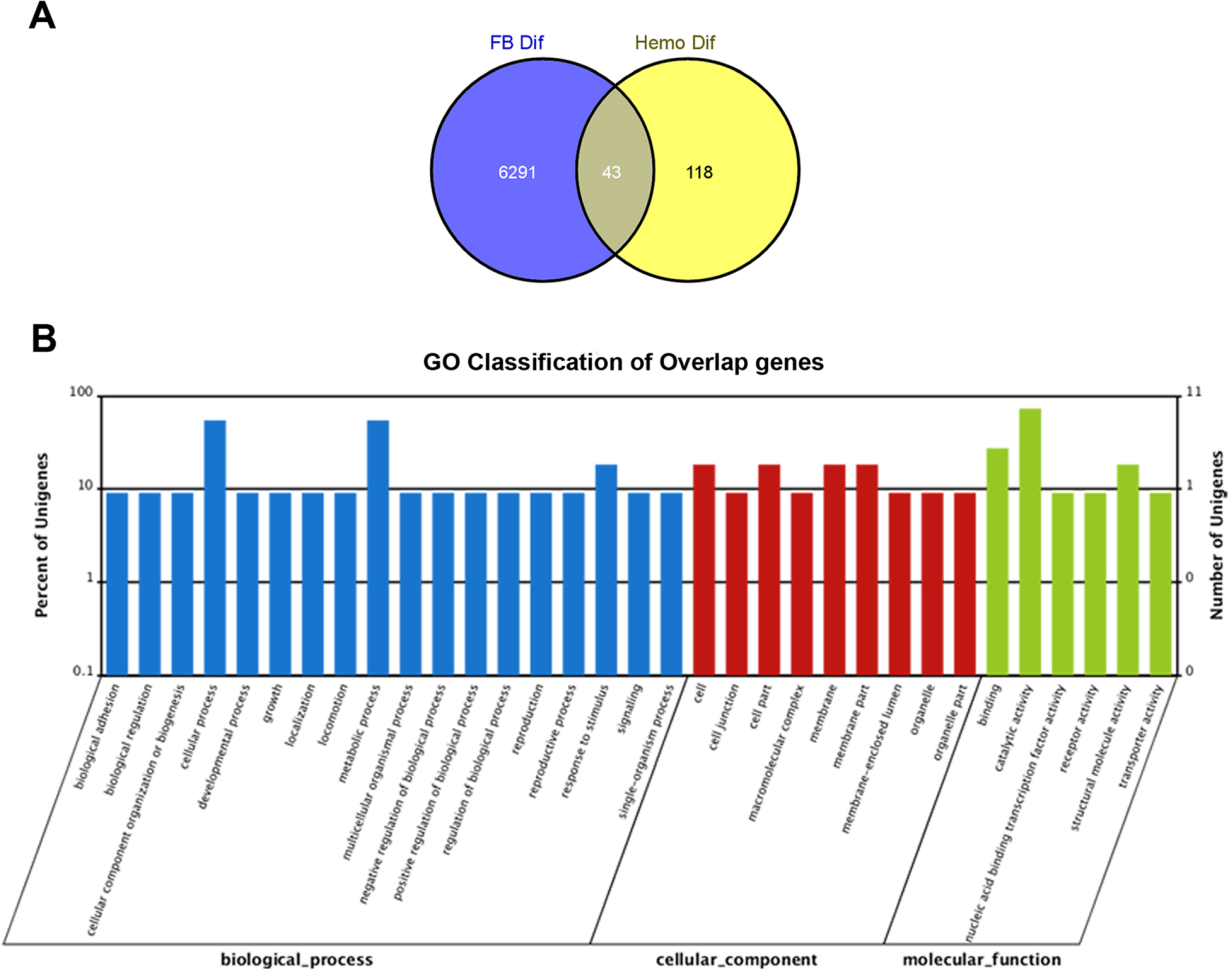
**a** Overlap between differentially expressed genes in the locust fat body and in hemocytes after *M. acridum* infection. **b** GO classification of fat body and hemocyte overlapping DEG dataset

GO annotation analyses resulted in 128 (69.6 %) and 4700 (74.2 %) differential expressed unigenes that could be mapped to the term “biological process” in the hemocyte and fat body datasets, respectively (Fig. 4a & b). For hemocytes, enriched expressed genes including those involved in nitric oxide biosynthesis, cellular amino acid biosynthesis, cold acclimation, organic acid biosynthesis, carboxylic acid biosynthesis, cuticle development and the chitin-based cuticle molting cycle, small molecule biosynthesis (Table 2). In the fat body response, GO annotation indicated enrichment of transcripts involved in development and cell differentiation including; mRNA translation (e.g. negative regulation of oskar involved in germ-plasm formation), oocyte development, pole plasm assembly, cytoplasmic organization, and cell maturation (Table 2). Overlap between the DEGs (differential expressed genes) enriched in the fat body and hemocytes after exposure to *M. acridum* was small (Fig. 5a), with those GO annotated within cellular and metabolic processes having the maximum number differentially expressed unigenes, i.e. six. The six unigenes were: aspartate 1-decarboxylase, phosphoserine phosphatase, two proteophosphoglycan ppg4, protein tyrosine phosphatase and zinc finger protein 768 (Fig. 5b).

**Table 2:** GO enrichment analysis

Gene ontology term	Cluster frequency	Genome frequency of use	Corrected *P*-value
Biological process			
Hemocyte			
Nitric oxide biosynthetic process	2/29, 7 %	4/8538, <0.01 %	0.01231
Cellular amino acid biosynthetic process	4/29, 14 %	89/8538, 1.0 %	0.03972
Cold acclimation	2/29, 7 %	7/8538, 0.1 %	0.04282
Nitric oxide metabolic process	2/29, 7 %	7/8538, 0.1 %	0.04282
Response to cold	2/29, 7 %	9 out of 8538 genes, 0.1 %	0.07310
Organic acid biosynthetic process	4/29, 14 %	128/8538, 2 %	0.15838
Carboxylic acid biosynthetic process	4/29, 14 %	128/8538, 2 %	0.15838
Cuticle development involved in chitin-based cuticle molting cycle	2/29, 7 %	18/8538, 0.2 %	0.30486
Cellular amino acid metabolic process	5/29, 17 %	265/8538, 3 %	0.33062
Small molecule biosynthetic process	4/29, 14 %	174/8538, 2.0 %	0.49169
Single-organism biosynthetic process	4/29, 14 %	183/8538, 2 %	0.59005
Fat body			
Regulation of pole plasm oskar mRNA localization	11/893, 1 %	25/8538, 0.3 %	0.03332
Regulation of oocyte development	11/893, 1 %	27/8538, 0.3 %	0.08004
Negative regulation of oskar mRNA translation	5/893, 1 %	6/8538, 0.1 %	0.13125
Pole plasm assembly	11/893, 1 %	30/8538, 0.4 %	0.24967
Cytoplasm organization	11/893, 1 %	33/8538, 0.4 %	0.65908
Cell maturation	23/893, 3 %	104/8538, 1 %	0.71693
Oocyte anterior/posterior axis specification	13/893, 2 %	45/8538, 0.5 %	0.95554
Cellular component			
Hemocyte			
Integral to membrane	8/17, 47 %	893/6281, 14 %	0.03580
Intrinsic to membrane	8/17, 47 %	907/6281, 14 %	0.03976
Membrane	11/17, 65 %	2186/6281, 35 %	0.34152
Membrane part	8/17, 47 %	1322/6281, 21 %	0.44314
Extracellular region	3/17, 18 %	257/6281, 4 %	0.90284
Fat body			
Intracellular	548/659, 83 %	4915/6281, 78 %	0.19681
Intracellular part	523/659, 79 %	4660/6281, 74 %	0.22058
Proton-transporting ATP synthase complex	8/659, 1 %	23/6281, 0.4 %	0.56557
Respiratory chain	14/659, 2 %	58/6281, 0.9 %	0.73999
Cytosolic small ribosomal subunit	10/659, 2 %	35/6281, 0.6 %	0.83309
Molecular function			
Hemocyte			
Nitric-oxide synthase activity	2/31, 7 %	3/9203, 0 %	0.00213
Oxidoreductase activity, acting on paired donors, with incorporation or reduction of molecular oxygen, NAD(P)H as one donor, and incorporation of one atom of oxygen	2/31, 7 %	4/9203, 0 %	0.00426
FMN binding	2/31, 7 %	10/9203, 0.1 %	0.03158
NADP binding	2/31, 7 %	18/9203, 0.2 %	0.10560
Oxidoreductase activity, acting on paired donors, with incorporation or reduction of molecular oxygen	3/31, 10 %	79/9203, 0.9 %	0.14961
Transaminase activity	2/31, 7 %	27/9203, 0.3 %	0.23774
Transferase activity, transferring nitrogenous groups	2/31, 7 %	27/9203, 0.3 %	0.23774
Hydrolase activity, acting on ester bonds	6/31, 19 %	558/9203, 6 %	0.63056
Monooxygenase activity	2/31, 7 %	45/9203, 0.5 %	0.64579
Calmodulin binding	2/31, 7 %	46/9203, 0.5 %	0.67373
Structural constituent of cuticle	2/31, 7 %	49/9203, 0.5 %	0.76074
Phosphoric ester hydrolase activity	4/31, 13 %	280/9203, 3 %	0.89824
Fat body			
mRNA 3′-UTR binding	9/955, 1 %	19/9203, 0.2 %	0.02320
O-methyltransferase activity	5/955 genes, 1 %	9/9203 genes, 0.1 %	0.51422
Stearoyl-CoA 9-desaturase activity	3/955, 0.3 %	3/9203, 0 %	0.54600
Acyl-CoA desaturase activity	3/955, 0.3 %	3/9203, 0 %	0.54600
Nucleic acid binding	159/955, 17 %	1241/9203, 14 %	0.89676
mRNA 5′-UTR binding	5/955, 1 %	10/9203, 0.1 %	0.94087

Annotations of transcripts into the “cellular component” category resulted in 57 (31 %) and 2588 (41 %) unigenes placed into various subcategories from the hemocyte and fat body DEGs, respectively (Fig. 4a & b). In hemocytes, membrane proteins and processes dealing with membrane biogenesis were the most enriched differentially expressed genes (Table 2). In the fat body, general metabolism, including ATP (Adenosine triphosphate) synthase and respiratory chain enzymes, along with intracellular processes and ribosomal subunits were the top subcategories showing differential expression enrichment (Table 2). A small set of co-differentially expressed unigenes were identified which included aspartate 1-decarboxylase, zinc finger protein, and multidrug resistance protein 1, the former two of which were also identified in the development and cell differentiation GO analysis.

GO “molecular function” analysis could assign 1388 (22 %) and 48 (26 %) of unigenes derived from the fat body and hemocyte DGE sets, respectively (Fig. 4a & b). Within the molecular function, the most abundant subcategories in hemocyte DEG dataset were nitric oxide synthase, oxidoreductase, FMN (flavin mononucleotide) and NADP (nicotinamide adenine dinucleotide phosphate) binding proteins, transaminase, transferase, hydrolase, monooxygenase, calmodulin-binding, and cuticle structural proteins and enzymes. (Table 2). In the fat body, abundant GO terms subcategories were identified to include; mRNA 3′- and 5′-UTR binding, O-methyltransferase activity, stearoyl-CoA 9-desaturase activity, acyl-CoA desaturase activity, and nucleic acid binding (Table 2). The co-differentially expressed unigene set included functions within catalytic activity (8), binding (3) and structural molecule activity (2).

KEGG pathway enrichment analysis revealed genes homologous to those found in pathways corresponding to neural processes, e.g. Parkinson’s disease, and myotrophic lateral sclerosis (ALS), as well as mRNA surveillance, spliceosome, and oxidative phosphorylation as being the top five most abundantly populated differentially (control versus fungal infected) expressed pathways in the fat body dataset. KEGG analysis of the hemocyte DEG dataset indicated enrichment of genes involved in amino acid metabolism, e.g. arginine, proline, glycine, serine, threonine, and tyrosine. Co-differential expressed pathways found in both the fat body and hemocyte datasets included sugar metabolism and response, and immune and infection responses, and signal transduction pathways, e.g. protein tyrosine phosphatases (PTP, Unigene21232_All) that are part of the JAK/STAT pathway.

GO enrichment analysis and KEEG pathway analysis revealed that complex biological processes or metabolic pathways were affected in both hemocyte and fat body. Overall, two major categories of genes were activated in hemocyte after infection. The first involved pathways linked to release of humoral immune factors, e.g. nitric oxide synthesis related genes, including two nitric oxide synthase [45], GTP (Guanosine-5′-triphosphate) cyclohydrolase I [46] mitochondrial uncoupling protein 2 [47], and melanization related genes, like GTP cyclohydrolase 1 [48]. The second category encompassed cellular immune function, and included protein rhomboid, cdc42, lipopolysaccharide-induced tumor necrosis factor-alpha factor homolog, which are proteins involved in phagocytosis [49–51], etc. By contrast, some humoral innate immune related genes, like Caspase-1 precursor, were activated in the fat body (more detailed information is given in the Discussion section concerning identification of immune related genes). Meanwhile, energy metabolism and reproduction related genes were affected significantly in fat body.

### Identification of immunity-related genes

**Fig. 6:**
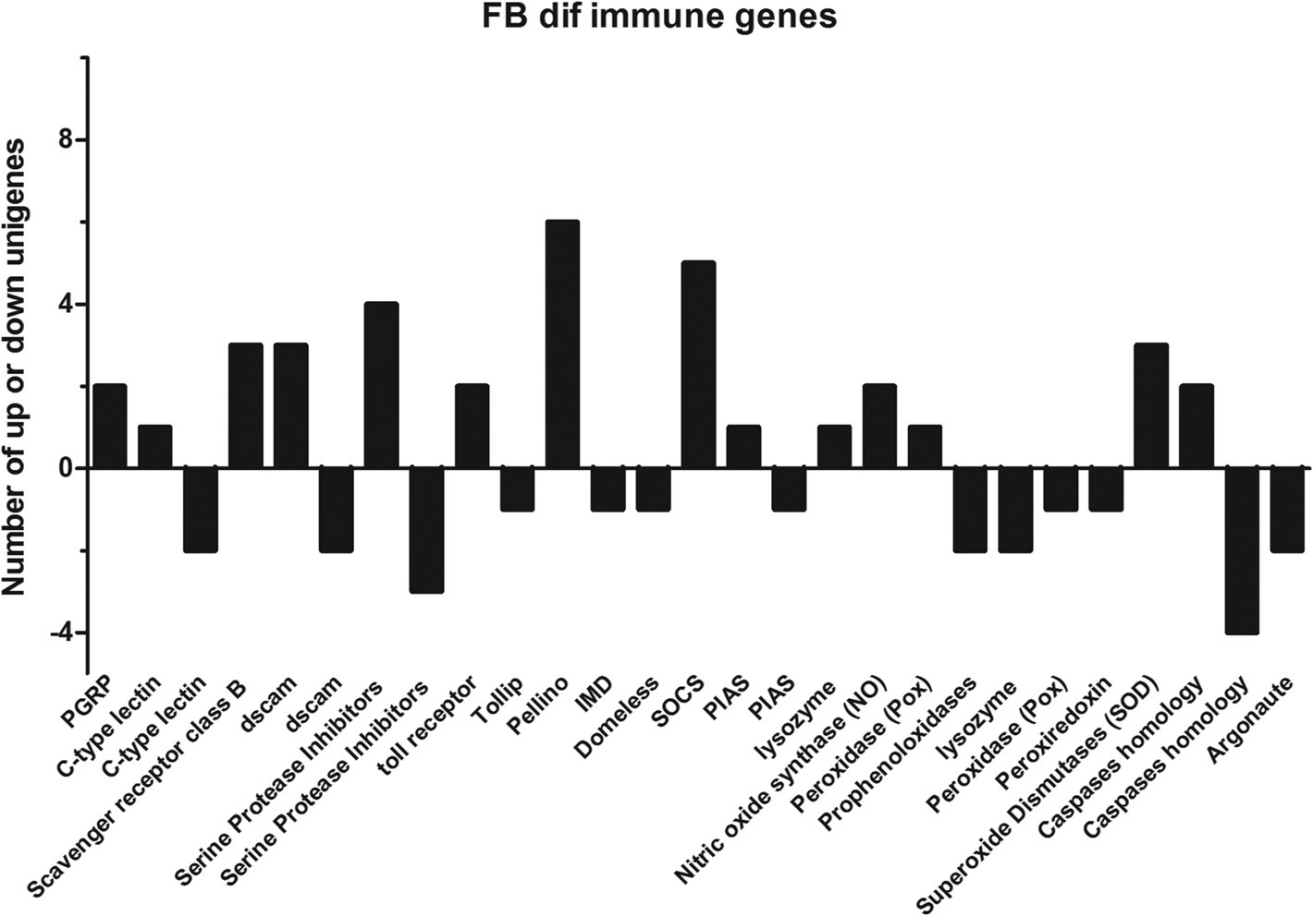
Functional classification of fat body DEGs in response to *M. acridum* infection

A number of locust immune related genes expressed in the fat body and hemocyte were identified using NCBI-BLAST analysis combined with conserved domain comparisons. Initial analyses were set to identify all immue related genes present in the transcriptome data. A second analysis was subsequently performed examining the differential expressed, i.e. control versus fungal infected, immune related gene sets in fat body and hemocyte subdatasets. In total 470 immune-related transcripts were identified. Immune-related responses found in the fat body encompassed genes involved in: (1) cellular pathogen recognition pathways and humoral immune reactions, (2) immune signal modulation and signal transduction, and (3) effectors and related-activties released into hemolymph. In the dataset obtained, 36 immune related genes were up-regulated and 23 were down-regulated in the fat body after infection (Fig. 6). In contrast, only three immune related genes, one lysozyme and two involved in NOS (nitric oxide synthases) pathway, were identifed in the hemocytes after infection, all of which were up-regulated. Within the fat body immune related dataset, DEGs included pathogen recognition and binding proteins, e.g. peptidoglycan recognition proteins (PGRPs, 2/14), C-type lectins (3/14), scavenger receptor class B protein (SCRB, 3/15), and immunoglobulin-like cell adhesion molecules (dscam proteins, 5/99); innate immune activation and suppression factors, e.g., serine protease inhibitors (serpins, 7/36), and prophenoloxidases (2/10); antimicrobial proteins, e.g. lysozymes (3/7); oxidative and stress responses, e.g. peroxidase (Pox, 2/37), superoxide dismutases (SODs, 3/12), and peroxiredoxin (1/11); cell death and inflammation, e.g. caspases (6/18); viral response and RNAi (RNA interference), e.g. argonaute (2/9), and NOS pathways, e.g. NOS synthase (2/5). In addition, members of the JAK/STAT immune realted signal trasnduction pathways were found including: *domeless* (1/2), *SOCS* (suppressor of cytokine signaling, 5/14), and *PIAS* (protein inhibitor of activated STAT, 2/6); as well as members of the Toll pathway, e.g. Toll receptors (2/21), *Tollip* (Toll Interacting Protein, 1/2), *Pellino* (6/12); and of the IMD pathway, e.g. *IMD* (1/2). A summary of the immune related gene identified is given in Additional file 1: Table S1.

### qRT-PCR analysis of immune-related genes

**Fig. 7:**
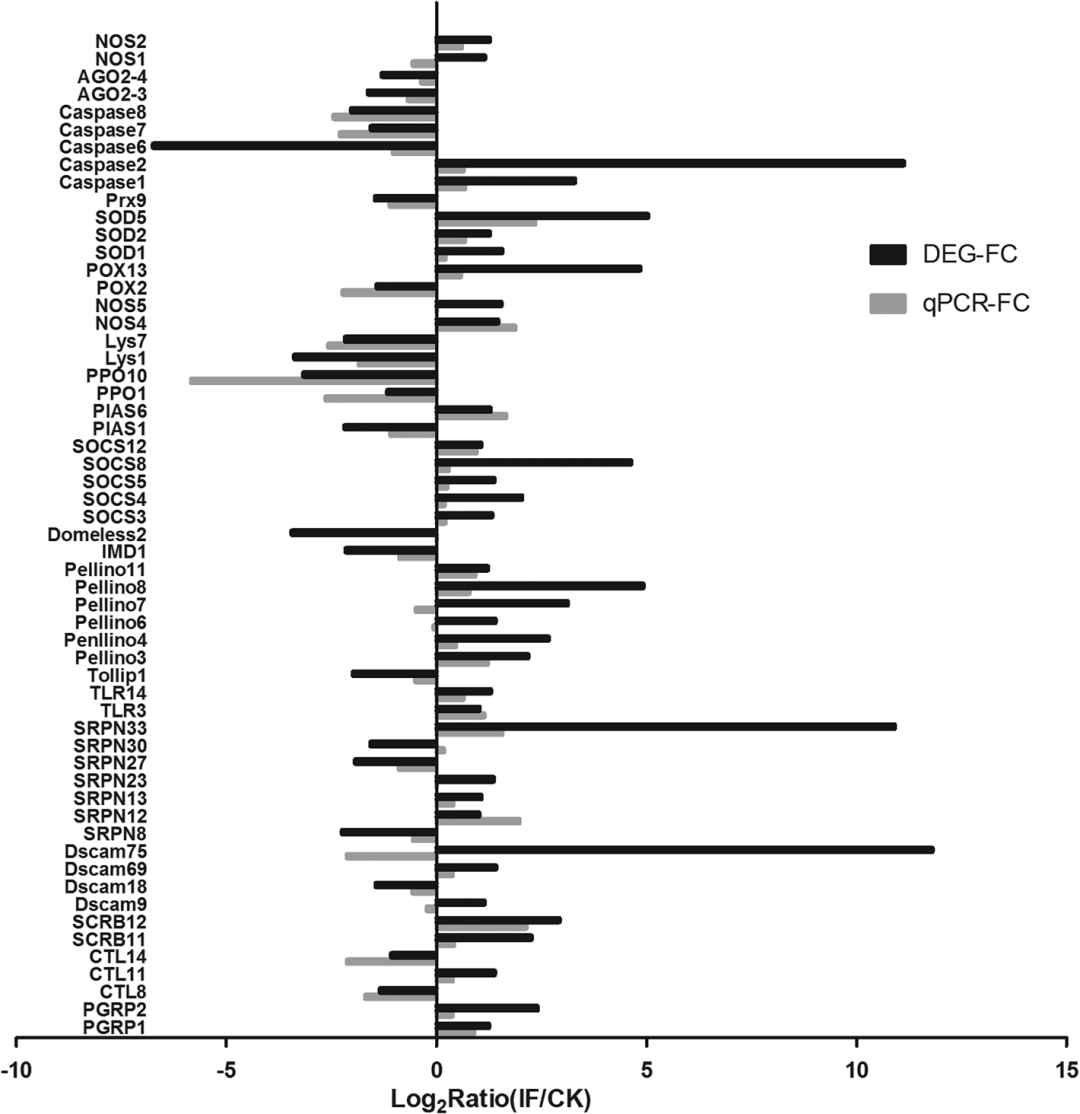
Q-RT-PCR analysis of DEGs and comparison to transcriptomics data

**Fig. 8:**
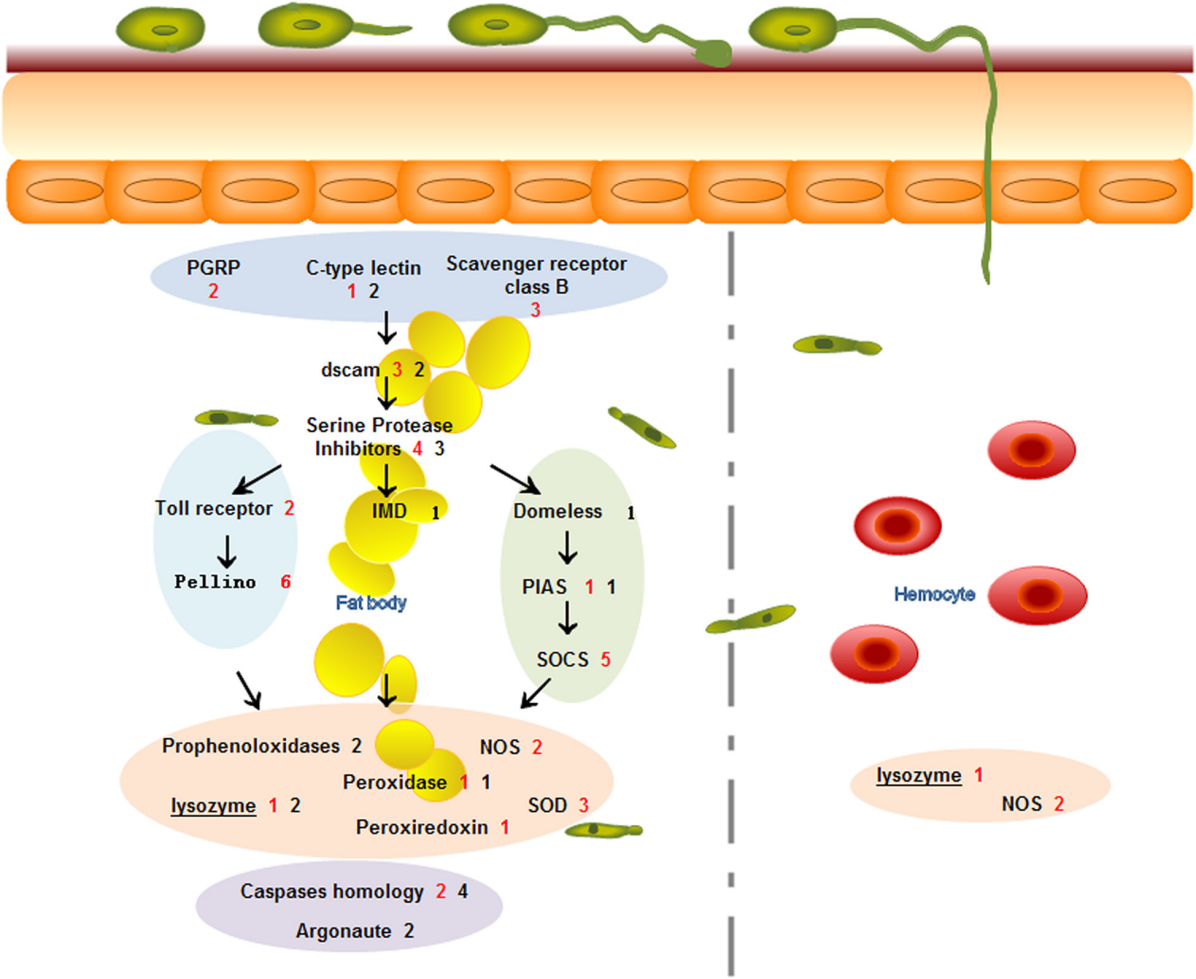
Summary of the major immune related DEGs identified in the fat body and in hemocytes to be up regulated (*red*) and down regulated (*black*) after *M. acridum* infection

qRT-PCR (Real-Time fluorescence quantitative PCR) was used to validate the differential expression of the immune related genes in the transcriptomic data. Sixty genes, including the 58 immune related DEGs identified in the fat body analysis and the three found in the hemocyte dataset were examined (one unigene is codifferentially expressed in fat body and hemocyte). For two genes (*Lys4*, *SCRB9*), primers could not be designed due to the limited sequence lengths isolated. Overall, 50/58 (86.2 %) were consistent with respect to expression between the transcriptomic and q-RT-PCR data (Fig. 7, Additional file 2: Table S2). With respect to the fat body DEGs, 7/55 (13 %) were found to be inconsistent with respect to expression between the transcriptomic and q-RT-PCR data (Fig. 7, Additional file 2: Table S2). These included *Dscam9* and *Dscam 75*, the serpins, *SRPN23* and *SRPN30*, *Pellino7* involved in the Toll pathway, *Domeless2*, part of the JAK/STAT pathway, and *NOS5*. OF the hemocyte DEGs, 1/2, namely the *NOS1* gene, showed inconsistent results comparing the transcriptomic and the q-RT-PCR data. A summary of the main immune-related pathways and specific genes up- or down- regulated in the fat body and in hemocytes, respectively after *M. acridum* infection is given in Fig. 8.

## Discussion

Insect responses to microbial pathogens begin at the cuticle and continue within the body of the organism with innate and induced responses. The insect fat body is the major organ mediating immune responses in insects, and it also governs organismal energy homeostasis including intermediary metabolism tissue, energy storage and utilization, and the synthesis of many hemolymph proteins and circulating metabolites [15]. Thus, changes in fat body gene expression in response to pathogens can result in a steep energy and/or reproductive cost [52]. Our results using the locust specific fungal pathogen, *M. acridum* showed similar changes in host gene expression broadly affecting reproductive and energy metabolism, effects consistent with other reports of such changes as a result or response to infection, acting as a potentially adaptive strategy to minimize or delay the spread of the infection [53]. In addition, phagocytic hemocytes, cells that freely float within the open circulatory system of insects, can consume foreign objects, including microbes, as well as initiate and/or contribute to encapsulation and melanization reactions that seek to limit the spread of invading microbes.

Limited reports [54], however, have focused on these two essential aspects of immune reactions, particularly examining specific tissue transcriptomic responses to pathogen attack. Here, we have examined the response of locust fat body and hemocytes to infection with the necrophytic insect pathogen, *M. acridum*. These data have allowed for the reconstruction of significant aspects of the locust immune pathways and the nature of the response to the fungal pathogen.

### Microbial recognition

Carbohydrate moeities appear to be important microbial antigens recognized by the insect innate immune system. These include often repeating polysaccharide units found on microbial surface strcutures and glycoproteins, e.g. lipopolysaccharide (LPS) on gram negative bacteria, peptidoglycan on gram positive bacteria, and high mannose and other surface glucans on yeast and filamentous fungi [55, 56]. Recognition initiates insect innate immune response and this step is mediated by pattern recognition molecules (pathogen sensors) that recognize conserved molecular patterns found on pathogens but presumably lacking in the host [57]. A variety of pattern recognition molecules have been described including peptidoglycan recognition protein (PGRP), β-1,3-glucan recognition protein (βGRPs), gram-negative binding proteins (GNBPs), Calcium-dependent (C-type) lectins (CTLs), and scavenger receptors (SCRs) [58]. Recognition, e.g. PGRP family members capable of distinguishing between various invading bacteria, acts upstream of Toll and Imd pathways to mediate. PGRPs can be categorized into short (PGRP-S) and long (PGRP-L) members, although all of these proteins have at least one conserved N-acetylmuramyl-alanine amidase-like domain [59].

Various insects have different PGRP repertiores; *Drosophila* has 13 PGRP genes encoding 19 proteins (due to alternative splicing) [60], mosquito (*Anopheles gambiae*); 7 PGRP genes encoding 9 proteins [61], and the silkworm, *Bombyx mori*, has 12 distinct PGRP genes [62]. Here, we identified 14 putative PGRP transcripts. Among them, two unigenes, PGRP-SA and PGRP-LC-like, were upregulated in the fat body in response to *M. acridum* infection. PGRP-SA is an essential component for activating the Toll pathway in *Drosophilia* [63]. PGRP-LC activates the Imd pathway [64]. These data indicate that in response to *M. acridum* both the Toll and Imd pathways have the potential to be activated.

The βGRP/GNBP proteins are another pattern recognition protein family found in most insects [65], including *Drosophila* (three genes) [66], *A. gambiae* (seven genes) [61], the honey bee, *Apis mellifera* (two genes) [10], tobacco hookworm, *Manduca sexta* (two genes) [67, 68], and in the beetle, *Tribolium castaneum* (three genes) [11]. βGRP proteins have high affinity for β-1,3-glucans found in fungal cell walls, while GNBPs bind to gram-negative or -positive bacteria [66]. All these members of this family contain a conserved N-terminal β-1,3-glucan-recognition domain for the detection of pathogens or parasites. Here, we found five GNBP genes (LmGNBP1-5) in the overall transcriptomic data, however none of these were differentially expressed after *M. acridum* infection in either the fat body or hemocytes. Our sequence alignment revealed that LmGNBP1 and 5 are identical to GNBP1 as named in Wang et al. [69] and LmGNBP3 is identical to GNBP2 [69] which was also the same as βGRP as reported by Zheng et al. [70]. In the Wang et al. report, LmGNBP1 and 5 (termed GNBP1) was initially expressed in hemocytes and was induced in the fat body 6 and 9 h after conidial injection into the hemocoel (note that this infection prtocol is different than ours in which infection was topical, i.e. followed the “natural route). We did not see any significant differential expression of LmGNBP1 and 5 although both LmGNBP1 and 5 are highly expressed (high FPKM values = 1619.8831 and 2318.7453, respectively) in heomcytes, with low expression in the fat body. Only a slight increase in expression of these genes were seen in the fat body after infection (FPKM from 11.26 to 16.05 and 4.4 to 4.7, respectively). These data suggest that LmGNBP1 and 5 are constitutively expressed in hemocytes, with only minor expression in the fat body consistant with the overall results of Wang et al. [69], althoug we did not see appreciable induction in the fat body. As noted above, this can be due to the different infection methods used. Regarding LmGNBP2 (termed βGRP in the Zheng et al. report [70], and GNBP3 in the Wang et al. report [69]), this gene was induced in hemocytes 8 and 12 h after inoculation of conidia, but no differences were seen earlier (4 h) or later (24, 48 and 72 h) post infection (as measured by q RT-PCR) in Zheng et al. paper. However, LmGNBP2 was detected in hemocyte in the absence of infection [70]. In our research, LmGNBP2 (= βGRP = GNBP3) simmilarly showed high expression in hemocytes and low expression in the fat body. As we did not examine intermediate time points, it is possible that a transient increase in LMGNBP2 was missed in our dataset. However, other have also reported constitutive but no significant differential expression of LmGNBP2 in any tissues [69], consistent with our results.

CTLs are a large family of carbohydrate binding proteins [71] that contribute to a number of inverberate immune responses, including microbial clearance [72], hemocyte nodule formation [73], activation of prophenoloxidase [74] and opsonisation [75]. In our study, 14 CTLs were identified in the transcriptome of the locust. Within the fat body differentially expressed gene set, *Lm*CTL8 and *Lm*CTL14 were significantly down regulated, whereas *Lm*CTL9 was up-regulated after fungal challenge.

Galectins are a phylogenetically ancient lectin family with evolutionary conserved carbohydrate binding domains [62]. Galectin-like proteins have been identified in *D. melanogaster* (*Dm*GALEs) and *A. gambiae* (*Ag*GALEs). In vitro experiments indicate that *Dm*GALEs can bind to ß-galactoside sugar [76], and that these proteins may play functions via facilitation of microbial recognition and/or phagocytosis [77]. Here, we identified five galectin-like proteins in the overall locust transcriptome, however, none of them appeared to be differentially expressed in either the fat body or in hemocytes after *M. acridum* infection.

Scavenger receptors are cell surface glycoproteins with structurally diverse transmembrane multidomains, divided into at least eight subfamilies (classes A to H, with A, B, and C, representing the major subfamilies) [78]. Class A scavenger receptors (SCRAs) are thought to function in phagocytic recognition of unoposonized and opsonsonnized microorganisms [79]. Class B SCRs participate in the phagocytosis of microbes [80], apoptotic-cell binding [81], etc. Class C SCRs are transmembrane or secreted multidomain proteins characterized by two complement-control protein (CCP) domains followed by a MAM domain (meprin A5 antigen and RPTP Mu), and usually a somatomedin-B-like (BO) domain. Drosophila has four class C SCRs subtypes (types I-IV), three of which has been shown to be involved in phagocytosis and innate immunity [82]. Our overall transcriptome dataset contained four unigenes corresponding to class A SCRs, 15 class B SCRs, and five class C SCRs. None of these genes, however, were found to be differentially expressed in either the fat body or in hemocytes after expsoure to *M. acridum*.

### Immunoglobulin-like genes

The Down syndrome cell adhesion molecule (DSCAM) is a immunoglobulin (Ig)-superfamily member with conserved architecture containing variable Ig and transmembrane domains [83]. *Dscam* is encoded by a single gene in *Drosophilia* but it can generate more than 18,000 isoforms via alternative splicing in *Drosophila* immune-competent cells [84]. DSCAM deficiency correlates with reduced phagocytotic uptake of bacteria [84] and differential splice variants of *Dscam* during exposure to different bacterial strain in *A. gambiae* has been reported [85] indicated that the complexity of dscam and its function in pathogen recognition. In the overall locust transcriptome dataset, we found ~100 *Dscam* unigenes (designated as DSCAM1-100), however, these likely represent splice variants. Among these, six unigenes showed significant differential expression in the locust fat body after *M. acridum* infection; three of which were upregualted (*Dscam9*, *69*, and *76*), with the latter >11-fold, whereas the other three unigenes were down regulated in the locust fat body (*Dscam14*, *18*, and *71*).

### Modulation

Once microbial pathogens are recognized by various receptor or recognition factors, different signalling cascades are activated via amplifaction or supressed via dampening. This typically occurs via activation/repression of extracellular proteases, via interplay between serine proteases and (serine) protease inhibitors (serpins). Clip domain serine proteases (CLIPs) are a large family of proteases that contain disulfide knotted clip domain(s), unique to arthropods. These domains are coupled to the active, C-terminal serine protease domain [86]. CLIPs are involved in melanization [87] and stimulation of the Toll pathway via activation of the phenoloxidase cascade by acting on the Toll-ligand Spätzle and other downstream proteins [88, 89]. *Drosophila* has 37 CLIP genes, *Anopheles* 41, *Bombyx* 15 and *Apis* 18. In *L. migratoria*, we ientified 34 potential CLIP unigenes, however, none were significantly differentially expressed after fungal infection.

Serpins (serine protease inhibitors) are a superfamily of proteins found in all higher eukaryotes as well as some viruses [90] that function through a unique suicide substrate-like inhibitory mechanism [91]. Serpins, typically composed of 350–400 amino acid residues, usually have a reactive center loop (RCL) located 30–40 residues from the C-terminal end that functions to bind the active site of specific proteases mimicking a substrate. Once an RCL moeity binds to its target protease, the protease can cleave the serpin at the scissile bond resulting in a covalent link of the serpin to the protease, thus blocking its activity [91]. Thirty-six *serpin* (*srpn*) transcripts were identified in the overall transcriptome dataset. Of these four were up-regulated and three down-regulated in the fat body after *M. acridum* infection. No srpn transcripts showed any significant differential expression in hemocytes after fungal infection.

### Signal transduction pathways: Toll, Imd, and JAK/STAT

After the extracellular signal derived from pathogen recognition has been modulated, and concomittant with activation of extracellualr defense pathways, the immune signal is transduced to target cells that activate cellular responses including the production of antimicrobial compounds and effectors. A number of immune related signal transduction pathways are present in insects including the Toll, Imd, and JAK/STAT pathways [10]. The Toll pathway is initiated by the input from PGRP/GNBP recognition of epitopes on the cell surfaces of bacteria or fungi [92]. Recognition (binding) activates extracellular serine protease cascades via cleavage of the extracellular cytokine pro-Spätzle to mature Spätzle. Mature Spätzle (C-terminal 106 amino acids) then binds to Toll receptors on insect cell surfaces, resulting in the recruitment (inside the cell) of the Tube/Myd88 complex that acts to stimulate the Pelle kinase [3]. Pelle phosphroylates Cactus, an inhibitory factor, resulting in its dissociation from the Cactus/Dorsal/Dif complex [93]. Dorsal and Dif are then free to translocate from the cytoplasm into the nucleus to activate the transcription of additional defense responses including the production of antimicrobial peptides. This is achieved via the activities of a host of additional proteins including Tollip, Pellino, TRAF2 (TNF receptor associated factor-2), and ECSIT (evolutionarily conserved signaling intermediate in Toll pathway), [94–96].

Spätzle shares high structural similarity with cystine-knot superfamily proteins, e.g. nerve growth factor (NGF) and Coagulogen [97]. Both *D. melanogaster* and *A. gambiae* contain six *Spätzle* genes, whereas *B. mori* has only three orthologs. However, thus far, it appears that only one *Spätzle* ortholog participates in immunity in *Drosophila* [98], and one *Spätzle* gene has been shown to be up regulated after bacterial infection in *A. gambiae*. In this study, four distinct *Spätzle* transcripts were identified, but none showed any significant differential expression in either the fat body or in hemocytes after fungal challenge. Toll and Toll-like receptors (TLR) are evolutionarily conserved proteins found from insects to mammals [99]. Toll/Toll-like receptors numbers vary from nine in *Drosophila*, to ten in *A. gambiae*, fourteen in *B, mori*, and five in *Apis mellifera* [62]. In *L. migratoria manilensis*, we have identified two *Toll* and twenty-one *Toll-like receptor* unigenes in the transcriptome dataset. Amongst these, expresssion of one Toll receptor was down-regulated, and expression of two *Toll-like receptors* were up-regulated in the locust fat body post fungal challenge. No changes were seen for any *Toll/Toll-like receptors* in hemocytes after fungal infection. Of downstream componenet of the Toll pathway, one *MyD88* (death domain containing myeloid differentiation factor 88) unigene was identified in the transcriptome dataset. Tube and Pelle are two additional proteins that share homology with MyD88 proteins (include Death domains). In our study, no *Tube* transcript was identified while two *Pelle* unigenes were found in the overall transcriptome dataset. None of the these genes, i.e. *MyD88/Pelle*, showed any differential expression in the fat body or in hemocytes after exposure to *M. acridum*. Twelve *Pellino* unigene transcripts were found in the overall locust transcriptome dataset, with expression of six *Pellino* unigenes up regulated in the locust fat body after *M. acridum* infection. Four *Cactus* and two Rel family transcription factor *Dorsal/Dif* unigenes were identified in the transcriptome dataset, none of which showed significant differential expression in the presence of *M. acridum*. Additonal intercellular components identified included *Tollip* (two unigenes), TRAF2 (TNF receptor associated factor-2) (two unigenes) and ECSIT (a single unigene). Amongst these, only one *Tollip* unigene was found to be significantly up regulated in infected locust fat bodies. Intruigingly, manipulation of the Toll system via genetic engineering of the insect pathogenic fungus *B. bassiana* to express a *Drosophila* serpin resulted in increased viruelnce towards a Lepidopteran host [100].

The Imd pathway has been shown to be induced by bacterial mesodiaminopimelic acid (DAP)-type peptidoglycans as well as their fungal counterparts, with weaker activation by lysine (Lys)-type peptidoglycan. Imd activation proceeds via epitope recognition by the transmembrane receptor PGRP-LC or by the receptor PGRP-LE [101]. Activated receptors then bind to the Imd protein which in turn interacts with the FADD (FAS-associated death-domain) protein. FADD associates with the apical caspase death-related Ced-3/Nedd2-like protein (DREDD) leading to the cleavage of Imd by DREDD. Proteolyzed Imd interacts with the *Drosophila* inhibitor of apoptosis-2 (dIAP-2) protein [102], which results in recruitment of downstream components, i.e. TAK1 (Transforming growth factor beta activated kinase-1) [103] and its adaptor TAB2 (TGF-beta activated kinase) [104]. Once recruited, TAK1 activates the IkB-Kinase (IKK) complex, resulting in the phosphorylation of the NF-kB protein (*Relish*) [105]. Further proteolytic cleavage of Relish by DREDD, results in a protein that can be translocated into the nucleus leading to transcriptional induction of the expression of defense compounds including antimicoribal peptides and other effectors [106, 107]. All essential components of the Imd pathway were identified in *L. migratoria manilensis* indicating that the Imd pathway is conserved in Orthoptera. In addition, three unigenes coding for Caspar proteins that act to suppress immune activation by blocking nuclear translocation of Relish were identified in the locust transcriptome dataset. Among this pathway, only IMD1 showed significant down-regulation in infected locust fat bodies.

The JAK/STAT pathway is known to respond to viral infections [108] and tissue damage [109]. Upon infection, hemocytes release cytokines, e.g. Unpaired-3 (Upd-3), that bind to the Domeless receptor [109] resulting in activation of JAK kinases (Janus kinase, e.g. encoded by the *Hopscotch* gene). Activated JAKs phosphorylates cytosolic STATs (signal transducers and activators of transcription) promoting their translocation to the nucleus where they act as transcriptional activators. With the exception of one component, *Upd*, all members of this pathway, including a single Hopscotch and two STAT unigenes, were identified in the locust transcriptome dataset. However, *Upd* has also not been found in *A. mellifera*, *A. gambiae*, *T. castaneum*, and *B. mori*, indicating that it may be a *Drosophila* specific ligand for the JAK/STAT Pathway. Two unigene transcripts for *Domeless* were identified in the locust, and one of them was significantly down regulated in the fat body after *M. acridum* infection. A number of additional JAK/STAT interacting proteins were identified. These included SOCS and PIAS genes. In this study, fourteen SOCS unigenes were identified, five of which were up regulated in the locust fat body after fungal challenge. Six unigene transcripts corresponding to PIAS genes were identifed, one of which (*Lm*PIAS1) up regulated, whereas another (*Lm*PIAS6) was down regulated in the locust fat body after *M. acridum* infection.

### Effectors

As part of the insect microbial defense response, pathogen recognition and signal modulation and transduction lead to the expression of various effectors that target the invading organism. These effectors stimulate phenoloxidase-dependent melanization, cellular apoptosis, and the production of antimicrobial compounds and peptides. PPO exists as an inactive zymogen in hemolymph. Recognition and regulation by serine proteases as described above, leads to PPO activation (conversion of PPO to PO) by an ultimate protease [110]. PO hydroxylates and oxidizes monophenols to quinones, resulting in several physiological consequences including toxicity to microbes, stimulation of melanin synthesis, faciliatation of the sequestreation of parasites, and wound healing [111]. Our overall transcriptome dataset contained ten PPO unigens, two of which were significantly down regulated in the locust fat body after *M. acridum* infection. Lysozyme (chicken or conventional type, C-type) is one of three classes (A, B, & C) of lysozymes found in the animal kingdom, with only the C-type found primarily in the Arthropoda and the phylum, Chordata. All lysozymes are characterized by their ability to hydrolyze the β-(1,4)-glycosidic bond between the alternating N-acetylmuramic acid (NAM) and N-acetylglucosamine (NAG) residues of peptidoglycan, the predominant cell wall polymer found in bacteria [112]. In this study, seven c-type lysozyme unigenes were identified in the *L. migratoria* transcriptome. Within the fat body response to *M. acridum* infection, two lysozyme unigenes were down-regulated and one up-regulated after fungal exposure. The latter lysozyme unigene was also up-regulated in hemocytes post-fungal infection. AMPs can be classified into three main groups; (a) proline, histidine or other amino acids-rich peptides, (b) linear peptides with α-helical conformations (e.g. cecropins and magainins), and (c) cyclic or open-ended cyclic peptides with pairs of cysteine disulfide bonds (e.g. defensins and protegrin). Unigenes corresponding to a defensin and a diptericin were identified in the overall transcriptome library, however, no differential expression of any of these was found in either the fat body or in hemocytes after *M. acridum* infection. This result is in accordance with previous report [69]. Oxygen-derived free radicals and nitric oxide are known to play important roles, both directly and/or as signalling molecules, during pathogen induced immune responses. For example, local production of free radicals is a critical component of acute-phase oxidative defense that targets invading microbes [113]. The production of these free radicals involved a variety of enzymes including, NOS, NADPH oxidases (NOX), peroxidases (POX), glutathione oxidases (GTX), superoxide dismutases (SOD), catalases, thioredoxins, thioredoxin reductases, and peroxiredoxins. In the overall transcriptome dataset we identified five NOS, seven NOX, 37 POX, 12 SOD, three catalase, 19 thioredoxin reductase, and 11 peroxiredoxin unigenes. Of these, two NOS unigene showed up regulation in the fat body, with two other NOS unigenes up regulated in hemocytes post-fungal infection. In addition, one POX and three SOD unigene were up-regulated whereas another POX and one peroxiredoxin unigene were down-regulated in the fat body.

### Other immune molecules

Caspases (cysteine aspartate-specific proteinases) both initiate and execute cellular apoptosis via cleavage of target proteins that lead to cell death. However, caspases are also involved in immune responses in non-apoptotic mechanisms, e.g. the Drosophila *DmDredd* and *DmIAP2* gene products are essential for Imd signaling [104, 114]. Caspases are also involved in insect antiviral responses [115]. Our transcriptome analysis of *L. migratoria manilensis* identified 20 caspase unigenes, five of which were down regulated and two of which were up regulated in the fat body of infected locusts. RNAi pathways play important roles not in normal development and in the targeted of foreign nucleic acids (primarily viral). Key components of the RNAi pathway include Dicer, enzyme that cleave RNA into siRNA (Small interfering RNA), and the RNA-induced silencing complex (RISC) that includes argonaute, which captures the siRNA, and targets gene transcription. Within the locust overall transcriptome dataset, four Dicer and nine argonaute unigenes were identified. Two argonaute unigenes were identified as being down regulated in the fat body of infected locusts, with no transcripts of any RNAi components identified found to be significantly differential expressed in hemocytes post *M. acridum* infection. However, glucose dehydrogenase was found to be a locust differential expressed protein in hemocyte in response to *M. acridum* that has been shown to be involved in encapsulation [116], and protein spinster homolog one induced caspase-independent autophagic cell death, factors which might facilitates removal of dead hemocyte after infection.

## Conclusion

Significant aspects of the locust physiology, including the main immune pathways were identified in the overall transcriptome analysis performed. By examining the reponse to infection of the two major organs or tissues involved in the immune response, namely the fat body and hemocytes, a set of differentially expressed genes were identified in each tissue. As *M. acridum* is a specific fungal pathogen of locusts, these data indicate that the locust attempts to mount a challegene to the fungal infection. The most significant responses, in terms of changes in gene expression levels, were found in the fat body, with hemocytes showing altered expression of only three genes (*Lm*Lys4, *Lm*NOS1, *Lm*NOS2). This may not be too surprising, as hemocytes are general scavengers, primed to target any invading microbe, depedning upon proper stimulation. In contrast, the fat body is capable of responding to specific threats via induction and signaling pathways to turn on the production of specific effectors, e.g. lysozyme and free radical production. Locust pathways for melanization, phagocytosis, and encapsulation were identified, however none of these were enough to thwart the fungus, indicating that it has evolved mechanisms for overcoming these defenses. These data establish the groundwork for further exploration of locust immune responses and the identification of potential targets for locust control.

## Additional files

Additional file 1: Table S1. Summary of the putative immunity-related unigenes identified in *Locusta migratoria manilensis*. (DOCX 65 kb) Additional file 2: Table S2. qRT-PCR primers. (DOCX 16 kb)

## Abbreviations

ALS: Myotrophic lateral sclerosis
AMPs: Anti-microbial peptides
ATP: Adenosine triphosphate
BO: Somatomedin-B-like
Caspases: Cysteine aspartate-specific proteinases
CCP: Complement-control protein
CLIPs: Clip domain serine proteases
COG: Cluster of orthologous groups
CTLs: Calcium-dependent (C-type) lectins
DEGs: Differential expressed genes
dIAP-2: Drosophila inhibitor of apoptosis-2
DREDD: Death-related Ced-3/Nedd2-like protein
DSCAM: Down syndrome cell adhesion molecule
ECSIT: Evolutionarily conserved signaling intermediate in Toll pathway
EST: Expressed sequenced tag
FADD: FAS-associated death-domain
FDR: False discovery rate
FMN: Flavin mononucleotide
FPKM: Fragments per kb per million fragments
GNBPs: Gram-negative binding proteins
GO: Gene ontology
GTP: Guanosine-5′-triphosphate
GTX: Glutathione oxidases
IKK: IkB-Kinase
Imd: Immune deficiency
IPM: Integrated pest management
JAK/STAT: Janus kinase/signal transduction and activator of transcription
KEGG: Kyoto Encyclopedia of genes and genomes
LoPS: Locust physiological saline solution
LPS: Lipopolysaccharide
MAM: Meprin A5 antigen and RPTP Mu
NADP: Nicotinamide adenine dinucleotide phosphate
NAG: N-acetylglucosamine
NAM: N-acetylmuramic acid
NOS: Nitric oxide synthases
NOX: NADPH oxidases
Nr: Non-redundant protein databases
PAMP: Pathogen-associated molecular pattern
PGRPs: Peptidoglycan recognition proteins
Pox: Peroxidase
PPO: Prophenoloxidase
PPRs: Pattern recognition receptors
PTP: Protein tyrosine phosphatases
qRT-PCR: Real-time fluorescence quantitative PCR
RCL: Reactive center loop
RISC: RNA-induced silencing complex
RNAi: RNA interference
RNI: Reactive intermediates nitron
SCRAs: Class A scavenger receptors
SCRB: Scavenger receptor class B protein
SCRs: Scavenger receptors
SDA: Sabouraud dextrose agar
serpins: Serine protease inhibitors
siRNA: Small interfering RNA
SOCS: Suppressor of cytokine signaling
SODs: Superoxide dismutases
SRA: Sequence read archive
TAB2: TGF-beta activated kinase 1
TAK1: Transforming growth factor beta activated kinase-1
TOLLIP: Toll interacting protein
TRAF2: TNF receptor associated factor-2
Upd-3: Unpaired-3
βGRP: β-1,3-glucan recognition protein
