# Supplementary material for: Comparative transcriptomic analysis of immune responses of the migratory locust, Locusta migratoria, to challenge by the fungal insect pathogen, Metarhizium acridum
Source: BMC Genomics. 2015 Oct 26;16:867. doi: 10.1186/s12864-015-2089-9 (PMC4624584; doi:10.1186/s12864-015-2089-9)
Supplement: Additional file 1: Table S1. — Summary of the putative immunity-related unigenes identified in Locusta migratoria manilensis. (DOCX 65 kb) [file 12864_2015_2089_MOESM1_ESM.docx]

**Additional file 1: Table S1** Summary of the putative immunity-related unigenes identified in *Locusta migratoria manilensis*.

| **Designated name** | **Unigene ID** | | | **FB log_2_ratio** | **Hemo log_2_ratio** | **Nucleotide length (nt)** | **Protein length (aa)** | **E value** | **Best Identity** |
| --- | --- | --- | --- | --- | --- | --- | --- | --- | --- |
| **Recognition** |  | | |  |  |  |  |  |  |
| **PGRP** |  | | |  |  |  |  |  |  |
| *LmPGRP1* | CL1449.Contig1_All | | | 1.2521 |  | 2210 | 186 | 1.00E-104 | 100 |
| *LmPGRP2* | CL2125.Contig2_All | | | 2.4027 |  | 1751 | 158 | 7.00E-36 | 48 |
| *LmPGRP3* | CL2125.Contig1_All | | | |  | 1693 | 158 | 7.00E-36 | 48 |
| *LmPGRP4* | CL1449.Contig2_All | | | |  | 2255 | 164 | 5.00E-58 | 70 |
| *LmPGRP5* | CL3824.Contig1_All | | | |  | 942 | 177 | 6.00E-29 | 34 |
| *LmPGRP6* | CL25.Contig3_All | | | |  | 4207 | 383 | 8.00E-36 | 28 |
| *LmPGRP7* | Unigene6222_All | | | |  | 1287 | 223 | 3.00E-32 | 30 |
| *LmPGRP8* | CL1038.Contig2_All | | | |  | 2616 | 87 | 2.00E-17 | 49 |
| *LmPGRP9* | CL1038.Contig1_All | | | |  | 434 | 87 | 4.00E-19 | 51 |
| *LmPGRP10* | Unigene14842_All | | | |  | 1474 | 66 | 4.00E-20 | 71 |
| *LmPGRP11* | CL3824.Contig2_All | | | |  | 671 | 150 | 3.00E-18 | 31 |
| *LmPGRP12* | Unigene27164_All | | | |  | 581 | 141 | 2.00E-16 | 35 |
| *LmPGRP13* | CL2493.Contig2_All | | | |  | 787 | 51 | 4.00E-12 | 61 |
| *LmPGRP14* | CL2493.Contig1_All | | | |  | 761 | 51 | 3.00E-12 | 61 |
| **βGNBP** |  | | |  |  |  |  |  |  |
| *Lm*GNBP1 | CL2777.Contig1_All | | | |  | 1515 | 455 | 2.00E-88 | 99 |
| *Lm*GNBP2 | CL2777.Contig2_All | | | |  | 1913 | 496 | 3.00E-94 | 100 |
| *Lm*GNBP3 | Unigene29531_All | | | |  | 1576 | 278 | 5.00E-51 | 41 |
| *Lm*GNBP4 | Unigene1829_All | | | |  | 656 | 155 | 1.00E-91 | 100 |
| *Lm*GNBP5 | Unigene10719_All | | | |  | 1318 | 149 | 6.00E-37 | 54 |
| **C-type lectin** |  | | |  |  |  |  |  |  |
| *Lm*CTL1 | CL372.Contig1_All | | | |  | 713 | 154 | 1.00E-85 | 91 |
| *Lm*CTL2 | Unigene20460_All | | | |  | 830 | 221 | 1.00E-120 | 90 |
| *Lm*CTL3 | Unigene15581_All | | | |  | 787 | 229 | 9.00E-110 | 79 |
| *Lm*CTL4 | CL2935.Contig2_All | | | |  | 3306 | 206 | 3.00E-61 | 54 |
| *Lm*CTL5 | CL2935.Contig1_All | | | |  | 1465 | 206 | 1.00E-61 | 54 |
| *Lm*CTL6 | Unigene9282_All | | | |  | 1551 | 295 | 2.00E-69 | 49 |
| *Lm*CTL7 | Unigene9243_All | | | |  | 923 | 220 | 8.00E-47 | 42 |
| *Lm*CTL8 | Unigene12862_All | | | -1.3498 |  | 1066 | 230 | 1.00E-50 | 42 |
| *Lm*CTL9 | CL3438.Contig1_All | | | |  | 1134 | 237 | 5.00E-34 | 41 |
| *Lm*CTL10 | Unigene18832_All | | | |  | 2850 | 194 | 2.00E-36 | 39 |
| *Lm*CTL11 | CL3438.Contig2_All | | | 1.3871 |  | 1193 | 256 | 4.00E-31 | 37 |
| *Lm*CTL12 | CL920.Contig1_All | | | |  | 2536 | 276 | 6.00E-29 | 32 |
| *Lm*CTL13 | CL1664.Contig8_All | | | |  | 1396 | 252 | 2.00E-25 | 30 |
| *Lm*CTL14 | Unigene20047_All | | | -1.0741 |  | 925 | 133 | 3.00E-12 | 29 |
| **Galectins** |  | | |  |  |  |  |  |  |
| *Lm*Galectin1 | Unigene6535_All | | | |  | 2154 | 300 | 6.00E-59 | 41 |
| *Lm*Galectin2 | Unigene31513_All | | | |  | 399 | 131 | 6.00E-34 | 50 |
| *Lm*Galectin3 | CL4390.Contig3_All | | | |  | 275 | 87 | 2.00E-16 | 36 |
| *Lm*Galectin4 | Unigene1713_All | | | |  | 705 | 99 | 2.00E-17 | 53 |
| *Lm*Galectin5 | Unigene33897_All | | | |  | 225 | 73 | 1.00E-24 | 66 |
| **Scavenger receptor class A** | | | |  |  |  |  |  |  |
| *Lm*SCRA1 | Unigene8455_All | | | |  | 2523 | 183 | 3.00E-71 | 36 |
| *Lm*SCRA2 | Unigene11930_All | | | |  | 218 | 67 | 1.00E-22 | 45 |
| *Lm*SCRA3 | Unigene17700_All | | | |  | 976 | 230 | 3.00E-107 | 76 |
| *Lm*SCRA4 | Unigene1103_All | | | |  | 569 | 99 | 2.00E-14 | 39 |
| **Scavenger receptor class B** | | | |  |  |  |  |  |  |
| *Lm*SCRB1 | Unigene4438_All | | | |  | 2378 | 474 | 5.00E-109 | 41 |
| *Lm*SCRB2 | Unigene8672_All | | | |  | 4236 | 537 | 7.00E-74 | 69 |
| *Lm*SCRB3 | Unigene15148_All | | | |  | 1021 | 332 | 7.00E-64 | 38 |
| *Lm*SCRB4 | Unigene2588_All | | | |  | 2909 | 434 | 4.00E-92 | 38 |
| *Lm*SCRB5 | Unigene18727_All | | | |  | 4928 | 521 | 2.00E-159 | 52 |
| *Lm*SCRB6 | CL2036.Contig1_All | | | |  | 2103 | 461 | 2.00E-94 | 37 |
| *Lm*SCRB7 | Unigene4268_All | | | |  | 2522 | 471 | 2.00E-81 | 35 |
| *Lm*SCRB8 | CL4434.Contig2_All | | | |  | 3263 | 446 | 1.00E-132 | 51 |
| *Lm*SCRB9 | Unigene19132_All | | | 1.8883 |  | 528 | 171 | 7.00E-56 | 57 |
| *Lm*SCRB10 | CL4434.Contig1_All | | | |  | 3132 | 436 | 8.00E-127 | 50 |
| *Lm*SCRB11 | Unigene16315_All | | | 2.2615 |  | 363 | 120 | 4.00E-31 | 47 |
| *Lm*SCRB12 | Unigene13774_All | | | 2.9289 |  | 734 | 107 | 1.00E-17 | 42 |
| *Lm*SCRB13 | Unigene5235_All | | | |  | 1147 | 277 | 1.00E-101 | 65 |
| *Lm*SCRB14 | CL2036.Contig2_All | | | |  | 703 | 186 | 1.00E-35 | 42 |
| *Lm*SCRB15 | Unigene17553_All | | | |  | 1510 | 286 | 1.00E-101 | 64 |
| **Scavenger receptor class C** | | | |  |  |  |  |  |  |
| *Lm*SCRC1 | Unigene5426_All | | | |  | 807 | 251 | 8.00E-64 | 46 |
| *Lm*SCRC2 | Unigene5957_All | | | |  | 2592 | 761 | 2.00E-91 | 67 |
| *Lm*SCRC3 | CL421.Contig2_All | | | |  | 10899 | 3549 | 4.00E-155 | 79 |
| *Lm*SCRC4 | CL1282.Contig2_All | | | |  | 3931 | 733 | 7.00E-29 | 67 |
| *Lm*SCRC5 | CL1282.Contig1_All | | | |  | 3877 | 751 | 7.00E-29 | 65 |
| **dscam** |  | | |  |  |  |  |  |  |
| *Lm*DSCAM1 | Unigene19970_All | | | |  | 290 |  | 3.00E-13 | 39 |
| *Lm*DSCAM2 | Unigene4352_All | | | |  | 2662 | 161 | 0 | 39 |
| *Lm*DSCAM3 | Unigene4431_All | | | |  | 1028 | 268 | 5.00E-167 | 44 |
| *Lm*DSCAM4 | Unigene31701_All | | | |  | 1147 | 327 | 9.00E-77 | 38 |
| *Lm*DSCAM5 | Unigene10670_All | | | |  | 4575 | 1212 | 0 | 28 |
| *Lm*DSCAM6 | CL4003.Contig2_All | | | |  | 16442 | 192 | 0 | 33 |
| *Lm*DSCAM7 | CL4003.Contig1_All | | | |  | 16499 | 230 | 0 | 33 |
| *Lm*DSCAM8 | Unigene12624_All | | | |  | 1826 | 237 | 3.00E-48 | 34 |
| *Lm*DSCAM9 | CL1640.Contig2_All | | | 1.1468 |  | 4385 | 619 | 0 | 28 |
| *Lm*DSCAM10 | CL1640.Contig1_All | | | |  | 3271 | 636 | 0 | 28 |
| *Lm*DSCAM11 | Unigene10468_All | | | |  | 14781 | 315 | 0 | 32 |
| *Lm*DSCAM12 | CL1213.Contig5_All | | | |  | 9311 | 456 | 0 | 35 |
| *Lm*DSCAM13 | CL1213.Contig4_All | | | |  | 8969 | 140 | 0 | 35 |
| *Lm*DSCAM14 | CL1213.Contig3_All | | | -1.8255 |  | 9119 | 433 | 0 | 35 |
| *Lm*DSCAM15 | CL1213.Contig1_All | | | |  | 9113 | 140 | 0 | 35 |
| *Lm*DSCAM16 | CL427.Contig6_All | | | |  | 24499 | 327 | 0 | 28 |
| *Lm*DSCAM17 | CL848.Contig4_All | | | |  | 6341 | 167 | 5.00E-146 | 26 |
| *Lm*DSCAM18 | CL848.Contig2_All | | | -1.4451 |  | 8012 | 247 | 2.00E-159 | 27 |
| *Lm*DSCAM19 | CL848.Contig1_All | | | |  | 6515 | 106 | 2.00E-151 | 26 |
| *Lm*DSCAM20 | CL3436.Contig2_All | | | |  | 12849 | 93 | 5.00E-103 | 28 |
| *Lm*DSCAM21 | CL3436.Contig1_All | | | |  | 8216 | 2679 | 0 | 28 |
| *Lm*DSCAM22 | Unigene4502_All | | | |  | 1397 | 410 | 0 | 39 |
| *Lm*DSCAM23 | CL427.Contig5_All | | | |  | 10673 | 1221 | 0 | 27 |
| *Lm*DSCAM24 | CL173.Contig5_All | | | |  | 2587 | 831 | 0 | 26 |
| *Lm*DSCAM25 | CL173.Contig14_All | | | |  | 9993 | 433 | 0 | 40 |
| *Lm*DSCAM26 | CL173.Contig10_All | | | |  | 2940 | 480 | 2.00E-54 | 26 |
| *Lm*DSCAM27 | CL173.Contig15_All | | | |  | 10167 | 433 | 0 | 40 |
| *Lm*DSCAM28 | CL173.Contig9_All | | | |  | 3114 | 480 | 5.00E-57 | 26 |
| *Lm*DSCAM29 | CL173.Contig7_All | | | |  | 2413 | 480 | 2.00E-54 | 26 |
| *Lm*DSCAM30 | CL173.Contig1_All | | | |  | 8043 | 433 | 0 | 40 |
| *Lm*DSCAM31 | CL173.Contig13_All | | | |  | 3756 | 1076 | 7.00E-74 | 26 |
| *Lm*DSCAM32 | CL173.Contig12_All | | | |  | 3582 | 422 | 5.00E-73 | 26 |
| *Lm*DSCAM33 | CL173.Contig6_All | | | |  | 3055 | 422 | 6.00E-73 | 26 |
| *Lm*DSCAM34 | CL173.Contig3_All | | | |  | 8685 | 292 | 0 | 40 |
| *Lm*DSCAM35 | CL3288.Contig2_All | | | |  | 2006 | 276 | 0 | 33 |
| *Lm*DSCAM36 | CL3288.Contig1_All | | | |  | 1669 | 276 | 0 | 33 |
| *Lm*DSCAM37 | CL173.Contig19_All | | | |  | 10827 | 292 | 0 | 40 |
| *Lm*DSCAM38 | CL173.Contig18_All | | | |  | 10653 | 292 | 0 | 40 |
| *Lm*DSCAM39 | CL173.Contig17_All | | | |  | 10725 | 292 | 0 | 40 |
| *Lm*DSCAM40 | CL173.Contig16_All | | | |  | 10551 | 300 | 0 | 40 |
| *Lm*DSCAM41 | CL173.Contig11_All | | | |  | 3774 | 1082 | 3.00E-73 | 26 |
| *Lm*DSCAM42 | CL173.Contig8_All | | | |  | 3600 | 428 | 3.00E-72 | 26 |
| *Lm*DSCAM43 | CL173.Contig4_All | | | |  | 3073 | 428 | 3.00E-72 | 26 |
| *Lm*DSCAM44 | CL173.Contig2_All | | | |  | 8703 | 292 | 0 | 40 |
| *Lm*DSCAM45 | Unigene1057_All | | | |  | 5645 | 1409 | 0 | 40 |
| *Lm*DSCAM46 | CL3777.Contig2_All | | | |  | 2147 | 215 | 0 | 30 |
| *Lm*DSCAM47 | CL4553.Contig2_All | | | |  | 1438 | 231 | 2.00E-121 | 33 |
| *Lm*DSCAM48 | CL4553.Contig1_All | | | |  | 1486 | 465 | 4.00E-129 | 33 |
| *Lm*DSCAM49 | Unigene728_All | | |  |  | 1209 | 193 | 3.00E-166 | 37 |
| *Lm*DSCAM50 | Unigene14753_All | | | |  | 3319 | 394 | 0 | 31 |
| *Lm*DSCAM51 | Unigene14612_All | | | |  | 3421 | 875 | 0 | 29 |
| *Lm*DSCAM52 | Unigene8509_All | | | |  | 3084 | 383 | 5.00E-27 | 29 |
| *Lm*DSCAM53 | Unigene6258_All | | | |  | 4894 | 1371 | 0 | 27 |
| *Lm*DSCAM54 | Unigene33866_All | | | |  | 353 | 117 | 2.00E-42 | 46 |
| *Lm*DSCAM55 | Unigene15118_All | | | |  | 2148 | 337 | 2.00E-86 | 29 |
| *Lm*DSCAM56 | Unigene13484_All | | | |  | 3195 | 130 | 1.00E-119 | 28 |
| *Lm*DSCAM57 | CL2642.Contig2_All | | | |  | 4637 | 351 | 0 | 28 |
| *Lm*DSCAM58 | Unigene17760_All | | | |  | 508 | 151 | 3.00E-45 | 35 |
| *Lm*DSCAM59 | Unigene18761_All | | | |  | 4403 | 483 | 0 | 26 |
| *Lm*DSCAM60 | CL1570.Contig1_All | | | |  | 5502 | 373 | 9.00E-172 | 29 |
| *Lm*DSCAM61 | Unigene36273_All | | | |  | 213 | 71 | 6.00E-27 | 56 |
| *Lm*DSCAM62 | CL1311.Contig1_All | | | |  | 2613 | 287 | 0 | 34 |
| *Lm*DSCAM63 | Unigene60_All | | |  |  | 5626 | 1090 | 0 | 27 |
| *Lm*DSCAM64 | Unigene4246_All | | | |  | 3045 | 422 | 9.00E-65 | 27 |
| *Lm*DSCAM65 | Unigene10784_All | | | |  | 801 | 237 | 3.00E-109 | 33 |
| *Lm*DSCAM66 | CL1136.Contig3_All | | | |  | 5939 | 156 | 0 | 32 |
| *Lm*DSCAM67 | CL1136.Contig1_All | | | |  | 6131 | 156 | 0 | 32 |
| *Lm*DSCAM68 | CL2143.Contig1_All | | | |  | 3003 | 886 | 0 | 26 |
| *Lm*DSCAM69 | CL736.Contig2_All | | | 1.4191 |  | 8578 | 105 | 0 | 24 |
| *Lm*DSCAM70 | CL736.Contig1_All | | | |  | 3462 | 1080 | 0 | 24 |
| *Lm*DSCAM71 | Unigene34883_All | | | |  | 293 | 86 | 3.00E-16 | 37 |
| *Lm*DSCAM72 | Unigene32913_All | | | |  | 220 | 64 | 9.00E-18 | 54 |
| *Lm*DSCAM73 | CL1136.Contig4_All | | | |  | 6710 | 1907 | 0 | 35 |
| *Lm*DSCAM74 | CL1136.Contig2_All | | | |  | 6518 | 1842 | 0 | 35 |
| *Lm*DSCAM75 | Unigene27388_All | | | 11.8061 |  | 428 | 105 | 6.00E-21 | 57 |
| *Lm*DSCAM76 | CL3141.Contig1_All | | | |  | 698 | 203 | 3.00E-82 | 33 |
| *Lm*DSCAM77 | Unigene8414_All | | | |  | 4194 | 897 | 0 | 32 |
| *Lm*DSCAM78 | Unigene37854_All | | | |  | 218 | 71 | 6.00E-21 | 46 |
| *Lm*DSCAM79 | Unigene22809_All | | | |  | 553 | 182 | 2.00E-47 | 32 |
| *Lm*DSCAM80 | Unigene12939_All | | | |  | 2982 | 188 | 4.00E-69 | 30 |
| *Lm*DSCAM81 | Unigene37726_All | | | |  | 232 | 68 | 9.00E-17 | 43 |
| *Lm*DSCAM82 | Unigene33532_All | | | |  | 243 | 81 | 6.00E-32 | 41 |
| *Lm*DSCAM83 | Unigene19512_All | | | |  | 660 | 217 | 4.00E-126 | 30 |
| *Lm*DSCAM84 | Unigene17236_All | | | |  | 1856 | 444 | 0 | 25 |
| *Lm*DSCAM85 | Unigene6515_All | | | |  | 1087 | 352 | 1.00E-106 | 30 |
| *Lm*DSCAM86 | Unigene612_All | | |  |  | 1058 | 197 | 1.00E-76 | 39 |
| *Lm*DSCAM87 | Unigene10616_All | | | |  | 7199 | 2105 | 0 | 31 |
| *Lm*DSCAM88 | Unigene764_All | | |  |  | 478 | 136 | 4.00E-61 | 30 |
| *Lm*DSCAM89 | Unigene8308_All | | | |  | 3361 | 743 | 1.00E-83 | 24 |
| *Lm*DSCAM90 | CL159.Contig3_All | | | |  | 4054 | 772 | 1.00E-84 | 24 |
| *Lm*DSCAM91 | CL1123.Contig2_All | | | |  | 3467 | 643 | 6.00E-81 | 28 |
| *Lm*DSCAM92 | Unigene18734_All | | | |  | 3720 | 915 | 5.00E-137 | 25 |
| *Lm*DSCAM93 | CL4684.Contig1_All | | | |  | 1643 | 389 | 2.00E-61 | 27 |
| *Lm*DSCAM94 | Unigene6719_All | | | |  | 3892 | 733 | 7.00E-99 | 25 |
| *Lm*DSCAM95 | Unigene15552_All | | | |  | 444 | 146 | 6.00E-61 | 33 |
| *Lm*DSCAM96 | CL580.Contig2_All | | | |  | 5656 | 455 | 0 | 21 |
| *Lm*DSCAM97 | CL580.Contig1_All | | | |  | 5422 | 434 | 0 | 21 |
| *Lm*DSCAM98 | Unigene10085_All | | | |  | 373 | 124 | 6.00E-67 | 32 |
| *Lm*DSCAM99 | Unigene12872_All | | | |  | 699 | 155 | 2.00E-41 | 26 |
| **Modulation** |  | | |  |  |  |  |  |  |
| **CLIP-domain serine protease** | | | |  |  |  |  |  |  |
| *Lm*CLIP1 | CL562.Contig2_All | | | |  | 2233 | 315 | 5.00E-54 | 39 |
| *Lm*CLIP2 | CL562.Contig1_All | | | |  | 2290 | 315 | 5.00E-54 | 39 |
| *Lm*CLIP3 | Unigene11117_All | | | |  | 2080 | 415 | 1.00E-104 | 47 |
| *Lm*CLIP4 | Unigene17000_All | | | |  | 1780 | 403 | 2.00E-78 | 42 |
| *Lm*CLIP5 | Unigene10766_All | | | |  | 2058 | 380 | 2.00E-70 | 39 |
| *Lm*CLIP6 | Unigene4663_All | | | |  | 1067 | 265 | 3.00E-51 | 41 |
| *Lm*CLIP7 | Unigene5180_All | | | |  | 547 | 182 | 1.00E-49 | 52 |
| *Lm*CLIP8 | Unigene8712_All | | | |  | 1502 | 309 | 1.00E-85 | 44 |
| *Lm*CLIP9 | Unigene15144_All | | | |  | 1084 | 211 | 6.00E-95 | 77 |
| *Lm*CLIP10 | Unigene15115_All | | | |  | 1593 | 304 | 4.00E-145 | 76 |
| *Lm*CLIP11 | Unigene4694_All | | | |  | 1478 | 362 | 1.00E-145 | 67 |
| *Lm*CLIP12 | Unigene17011_All | | | |  | 1852 | 270 | 1.00E-131 | 81 |
| *Lm*CLIP13 | Unigene29605_All | | | |  | 939 | 207 | 6.00E-56 | 54 |
| *Lm*CLIP14 | CL3790.Contig2_All | | | |  | 1589 | 455 | 1.00E-158 | 61 |
| *Lm*CLIP15 | CL3264.Contig2_All | | | |  | 1264 | 262 | 3.00E-46 | 55 |
| *Lm*CLIP16 | CL3264.Contig1_All | | | |  | 1501 | 262 | 4.00E-46 | 55 |
| *Lm*CLIP17 | Unigene15620_All | | | |  | 450 | 148 | 6.00E-35 | 49 |
| *Lm*CLIP18 | Unigene15023_All | | | |  | 2434 | 277 | 6.00E-103 | 64 |
| *Lm*CLIP19 | CL3790.Contig1_All | | | |  | 1261 | 411 | 2.00E-148 | 60 |
| *Lm*CLIP20 | Unigene5108_All | | | |  | 1392 | 269 | 1.00E-71 | 68 |
| *Lm*CLIP21 | Unigene15707_All | | | |  | 1211 | 134 | 1.00E-76 | 100 |
| *Lm*CLIP22 | Unigene20702_All | | | |  | 434 | 144 | 2.00E-66 | 82 |
| *Lm*CLIP23 | Unigene7519_All | | | |  | 410 | 133 | 6.00E-45 | 70 |
| *Lm*CLIP24 | CL3782.Contig1_All | | | |  | 1097 | 88 | 4.00E-43 | 90 |
| *Lm*CLIP25 | CL3782.Contig2_All | | | |  | 1033 | 71 | 1.00E-33 | 93 |
| *Lm*CLIP26 | Unigene20653_All | | | |  | 297 | 77 | 3.00E-16 | 57 |
| *Lm*CLIP27 | Unigene9731_All | | | |  | 223 | 67 | 2.00E-14 | 52 |
| *Lm*CLIP28 | Unigene21158_All | | | |  | 293 | 90 | 3.00E-17 | 47 |
| *Lm*CLIP29 | Unigene17496_All | | | |  | 402 | 128 | 5.00E-21 | 42 |
| *Lm*CLIP30 | Unigene6190_All | | | |  | 625 | 109 | 7.00E-57 | 95 |
| *Lm*CLIP31 | Unigene6975_All | | | |  | 1881 | 364 | 1.00E-81 | 46 |
| *Lm*CLIP32 | Unigene3395_All | | | |  | 419 | 139 | 1.00E-60 | 58 |
| *Lm*CLIP33 | Unigene14110_All | | | |  | 451 | 49 | 3.00E-10 | 35 |
| *Lm*CLIP34 | Unigene21474_All | | | |  | 317 | 83 | 8.00E-08 | 81 |
| **Serine Protease Inhibitors** | | | |  |  |  |  |  |  |
| *Lm*SRPN1 | Unigene2869_All | | | |  | 1846 | 383 | 3.00E-89 | 44 |
| *Lm*SRPN2 | CL46.Contig2_All | | | |  | 3091 | 325 | 1.00E-97 | 55 |
| *Lm*SRPN3 | Unigene14846_All | | | |  | 2557 | 393 | 1.00E-48 | 27 |
| *Lm*SRPN4 | CL46.Contig1_All | | | |  | 1563 | 325 | 6.00E-98 | 55 |
| *Lm*SRPN5 | CL2438.Contig5_All | | | |  | 1532 | 328 | 0 | 100 |
| *Lm*SRPN6 | Unigene17088_All | | | |  | 1353 | 325 | 2.00E-92 | 53 |
| *Lm*SRPN7 | CL1532.Contig1_All | | | |  | 1666 | 329 | 3.00E-87 | 53 |
| *Lm*SRPN8 | CL2438.Contig6_All | | | -2.2481 |  | 1643 | 328 | 0 | 100 |
| *Lm*SRPN9 | CL2438.Contig3_All | | | |  | 1589 | 328 | 0 | 100 |
| *Lm*SRPN10 | CL1532.Contig8_All | | | |  | 1530 | 329 | 2.00E-87 | 53 |
| *Lm*SRPN11 | Unigene5066_All | | | |  | 1326 | 323 | 4.00E-100 | 56 |
| *Lm*SRPN12 | CL1532.Contig2_All | | | 1.0204 |  | 1603 | 324 | 3.00E-92 | 53 |
| *Lm*SRPN13 | Unigene13436_All | | | 1.0681 |  | 1185 | 364 | 2.00E-72 | 42 |
| *Lm*SRPN14 | CL1532.Contig4_All | | | |  | 1425 | 324 | 2.00E-92 | 53 |
| *Lm*SRPN15 | CL1532.Contig3_All | | | |  | 1538 | 324 | 2.00E-92 | 53 |
| *Lm*SRPN16 | CL1532.Contig5_All | | | |  | 1470 | 324 | 2.00E-92 | 53 |
| *Lm*SRPN17 | CL1532.Contig7_All | | | |  | 1208 | 305 | 4.00E-87 | 53 |
| *Lm*SRPN18 | CL1532.Contig6_All | | | |  | 1278 | 324 | 2.00E-92 | 53 |
| *Lm*SRPN19 | CL683.Contig1_All | | | |  | 1416 | 392 | 5.00E-68 | 38 |
| *Lm*SRPN20 | Unigene28946_All | | | |  | 1088 | 324 | 5.00E-94 | 56 |
| *Lm*SRPN21 | Unigene28568_All | | | |  | 958 | 316 | 8.00E-72 | 45 |
| *Lm*SRPN22 | Unigene6745_All | | | |  | 2019 | 391 | 3.00E-97 | 44 |
| *Lm*SRPN23 | Unigene8566_All | | | 1.3679 |  | 1882 | 381 | 4.00E-80 | 41 |
| *Lm*SRPN24 | Unigene3378_All | | | |  | 2809 | 542 | 3.00E-65 | 30 |
| *Lm*SRPN25 | Unigene12042_All | | | |  | 890 | 223 | 5.00E-30 | 35 |
| *Lm*SRPN26 | Unigene28702_All | | | |  | 591 | 196 | 2.00E-46 | 46 |
| *Lm*SRPN27 | CL2438.Contig1_All | | | -1.937 |  | 352 | 117 | 2.00E-52 | 85 |
| *Lm*SRPN28 | CL2578.Contig1_All | | | |  | 1350 | 210 | 3.00E-15 | 27 |
| *Lm*SRPN29 | Unigene11131_All | | | |  | 1052 | 258 | 7.00E-43 | 36 |
| *Lm*SRPN30 | CL2438.Contig2_All | | | -1.5728 |  | 254 | 76 | 1.00E-24 | 76 |
| *Lm*SRPN31 | Unigene15992_All | | | |  | 267 | 75 | 1.00E-12 | 46 |
| *Lm*SRPN32 | Unigene21330_All | | | |  | 380 | 117 | 9.00E-15 | 38 |
| *Lm*SRPN33 | CL2578.Contig2_All | | | 10.9037 |  | 400 | 102 | 4.00E-24 | 49 |
| *Lm*SRPN34 | Unigene21453_All | | | |  | 278 | 92 | 3.00E-33 | 74 |
| *Lm*SRPN35 | Unigene11077_All | | | |  | 1444 | 96 | 8.00E-17 | 48 |
| *Lm*SRPN36 | Unigene26095_All | | | |  | 223 | 66 | 3.00E-11 | 48 |
| **Transduction** |  | | |  |  |  |  |  |  |
| **(Toll pathway)** |  | | |  |  |  |  |  |  |
| **Spatzle** |  | | |  |  |  |  |  |  |
| *LmSpz1* | CL1550.Contig3_All | | | |  | 1794 | 211 | 3.00E-22 | 49 |
| *LmSpz2* | CL1550.Contig2_All | | | |  | 1696 | 211 | 3.00E-22 | 49 |
| *LmSpz3* | CL1550.Contig1_All | | | |  | 1622 | 211 | 2.00E-22 | 49 |
| *LmSpz4* | Unigene13691_All | | | |  | 435 | 91 | 7.00E-17 | 56 |
| **Toll receptor** |  | | |  |  |  |  |  |  |
| *Lm*TLR1 | Unigene17159_All | | | |  | 3940 | 927 | 0 | 41 |
| *Lm*TLR2 | Unigene16733_All | | | |  | 3447 | 969 | 0 | 58 |
| *Lm*TLR3 | Unigene8510_All | | | 1.0278 |  | 3683 | 376 | 0 | 23 |
| *Lm*TLR4 | Unigene10617_All | | | |  | 2653 | 540 | 2.00E-141 | 46 |
| *Lm*TLR5 | Unigene17026_All | | | |  | 2227 | 630 | 0 | 55 |
| *Lm*TLR6 | Unigene19233_All | | | |  | 3119 | 580 | 7.00E-78 | 34 |
| *Lm*TLR7 | Unigene29607_All | | | |  | 1999 | 497 | 2.00E-21 | 23 |
| *Lm*TLR8 | Unigene29610_All | | | |  | 2067 | 406 | 1.00E-19 | 25 |
| *Lm*TLR9 | Unigene4555_All | | | |  | 2459 | 617 | 1.00E-45 | 28 |
| *Lm*TLR10 | Unigene30229_All | | | |  | 759 | 130 | 9.00E-11 | 32 |
| *Lm*TLR11 | Unigene20016_All | | | |  | 2028 | 374 | 6.00E-103 | 51 |
| *Lm*TLR12 | Unigene2476_All | | | |  | 2242 | 434 | 2.00E-100 | 44 |
| *Lm*TLR13 | CL2486.Contig3_All | | | |  | 632 | 178 | 1.00E-28 | 40 |
| *Lm*TLR14 | CL2486.Contig1_All | | | 1.2904 |  | 1815 | 180 | 4.00E-23 | 34 |
| *Lm*TLR15 | Unigene15288_All | | | |  | 1764 | 286 | 5.00E-91 | 53 |
| *Lm*TLR16 | CL2486.Contig2_All | | | |  | 255 | 83 | 3.00E-15 | 52 |
| *Lm*TLR17 | Unigene10936_All | | | |  | 1675 | 429 | 1.00E-42 | 29 |
| *Lm*TLR18 | CL990.Contig1_All | | | |  | 420 | 102 | 1.00E-10 | 33 |
| *Lm*TLR19 | CL3845.Contig3_All | | | |  | 593 | 133 | 2.00E-12 | 32 |
| *Lm*TLR20 | Unigene30124_All | | | |  | 729 | 109 | 3.00E-10 | 32 |
| *Lm*TLR21 | Unigene32907_All | | | |  | 349 | 105 | 1.00E-07 | 34 |
|  |  | | |  |  |  |  |  |  |
| **MyD88** |  | | |  |  |  |  |  |  |
| *Lm*MyD88 | Unigene15288_All | | | |  | 1764 | 286 | 5.00E-91 | 66 |
| **Pelle** |  | | |  |  |  |  |  |  |
| *Lm*Pelle1 | CL1980.Contig1_All | | | |  | 1658 | 470 | 4.00E-119 | 65 |
| *Lm*Pelle2 | CL1980.Contig2_All | | | |  | 1857 | 533 | 9.00E-147 | 65 |
| **Cactus** |  | | |  |  |  |  |  |  |
| *Lm*Cactus1 | CL1605.Contig3_All | | | |  | 2660 | 335 | 0 | 47 |
| *Lm*Cactus2 | CL1605.Contig2_All | | | |  | 2441 | 330 | 0 | 47 |
| *Lm*Cactus3 | CL1605.Contig1_All | | | |  | 2977 | 347 | 0 | 47 |
| *Lm*Cactus4 | CL1605.Contig4_All | | | |  | 3763 | 347 | 0 | 47 |
| **Dorsal/Dif** |  | | |  |  |  |  |  |  |
| *Lm*Dorsal1 | CL2728.Contig2_All | | | |  | 1746 | 468 | 1.00E-142 | 77 |
| *Lm*Dorsal2 | CL2728.Contig1_All | | | |  | 1828 | 461 | 7.00E-138 | 86 |
| **Tollip** |  | | |  |  |  |  |  |  |
| *Lm*Tollip1 | CL4192.Contig2_All | | | -1.9907 |  | 1237 | 274 | 2.00E-99 | 73 |
| *Lm*Tollip2 | CL4192.Contig1_All | | | |  | 1110 | 274 | 2.00E-99 | 73 |
| **Pellino** |  | | |  |  |  |  |  |  |
| *Lm*Pellino1 | Unigene19033_All | | | |  | 2602 | 434 | 0 | 82 |
| *Lm*Pellino2 | Unigene10368_All | | | |  | 5865 | 385 | 2.00E-97 | 56 |
| *Lm*Pellino3 | Unigene10367_All | | | 2.184 |  | 1685 | 385 | 4.00E-98 | 56 |
| *Lm*Pellino4 | Unigene10366_All | | | 2.6734 |  | 4432 | 385 | 1.00E-97 | 56 |
| *Lm*Pellino5 | CL210.Contig4_All | | | |  | 5635 | 385 | 2.00E-97 | 56 |
| *Lm*Pellino6 | CL210.Contig2_All | | | 1.4041 |  | 5414 | 385 | 2.00E-97 | 56 |
| *Lm*Pellino7 | Unigene10364_All | | | 3.1279 |  | 1591 | 237 | 1.00E-57 | 59 |
| *Lm*Pellino8 | CL4622.Contig2_All | | | 4.9298 |  | 1484 | 235 | 2.00E-57 | 60 |
| *Lm*Pellino9 | Unigene10359_All | | | |  | 2062 | 163 | 3.00E-33 | 59 |
| *Lm*Pellino10 | Unigene10361_All | | | |  | 878 | 121 | 8.00E-14 | 44 |
| *Lm*Pellino11 | Unigene10360_All | | | 1.2249 |  | 4466 | 92 | 6.00E-13 | 44 |
| *Lm*Pellino12 | Unigene10362_All | | | |  | 654 | 119 | 8.00E-14 | 44 |
| **TRAF2** |  | | |  |  |  |  |  |  |
| *Lm*TRAF2-1 | Unigene8523_All | | | |  | 1412 | 358 | 4.00E-81 | 61 |
| *Lm*TRAF2-2 | Unigene376_All | | |  |  | 1988 | 305 | 6.00E-147 | 33 |
| *Lm*TRAF2-3 | CL729.Contig2_All | | | |  | 2594 | 472 | 0 | 98 |
| **ECSIT** |  | | |  |  |  |  |  |  |
| *Lm*ECSIT | Unigene19062_All | | | |  |  | 360 | 1.00E-114 | 55 |
| **(Imd pathway)** |  | | |  |  |  |  |  |  |
| **IMD** |  | | |  |  |  |  |  |  |
| *Lm*IMD1 | CL3736.Contig2_All | | | -2.1566 |  | 1312 | 270 | 1.00E-150 | 28 |
| *Lm*IMD2 | CL3736.Contig1_All | | | |  | 1613 | 270 | 1.00E-150 | 28 |
| **FADD** |  | | |  |  |  |  |  |  |
| *Lm*FADD | Unigene2768_All | | | |  | 904 | 202 | 2.00E-19 | 36 |
| **Dredd** |  | | |  |  |  |  |  |  |
| *Lm*Dredd | Unigene146_All | | |  |  | 3906 | 532 | 2.00E-71 | 35 |
| **IAP2** |  | | |  |  |  |  |  |  |
| *Lm*IAP2-1 | Unigene9269_All | | | |  | 721 | 110 | 2.00E-25 | 51 |
| *Lm*IAP2-2 | Unigene18756_All | | | |  | 5003 | 1569 | 0 | 52 |
| *Lm*IAP2-3 | Unigene2548_All | | | |  | 1255 | 331 | 7.00E-102 | 27 |
| *Lm*IAP2-4 | Unigene6344_All | | | |  | 3589 | 546 | 0 | 43 |
| **Tak1** |  | | |  |  |  |  |  |  |
| *Lm*Tak1-1 | CL2381.Contig2_All | | | |  | 1678 | 476 | 0 | 71 |
| *Lm*Tak1-2 | CL2381.Contig1_All | | | |  | 695 | 152 | 2.00E-64 | 76 |
| *Lm*Tak1-3 | CL1819.Contig3_All | | | |  | 2252 | 212 | 1.00E-24 | 67 |
| *Lm*Tak1-4 | CL1819.Contig2_All | | | |  | 700 | 166 | 7.00E-25 | 67 |
| *Lm*Tak1-5 | CL1819.Contig1_All | | | |  | 760 | 177 | 9.00E-32 | 67 |
| **IKK** |  | | |  |  |  |  |  |  |
| *Lm*IKK | Unigene17201_All | | | |  | 2453 | 645 | 3.00E-152 | 56 |
| **Relish** |  | | |  |  |  |  |  |  |
| *Lm*Relish | Unigene18975_All | | | |  | 3176 | 950 | 0 | 54 |
| **caspar** |  | | |  |  |  |  |  |  |
| *Lm*capar1 | Unigene6576_All | | | |  | 2454 | 665 | 0 | 60 |
| *Lm*capar2 | Unigene2495_All | | | |  | 2326 | 451 | 2.00E-146 | 30 |
| *Lm*capar3 | Unigene32070_All | | | |  | 742 | 229 | 2.00E-15 | 34 |
| **(JAK/STAT pathway)** | | | |  |  |  |  |  |  |
| **UPD** |  | | |  |  |  |  |  |  |
| **Domeless** |  | | |  |  |  |  |  |  |
| *Lm*Domeless1 | CL2143.Contig1_All | | | |  | 3003 | 886 | 0 | 40 |
| *Lm*Domeless2 | CL2143.Contig2_All | | | -3.4396 |  | 3951 | 167 | 4.00E-52 | 47 |
| **Hopscotch** |  | | |  |  |  |  |  |  |
| *Lm*Hopscotch | Unigene6268_All | | | |  | 4090 | 1095 | 0 | 61 |
| **STAT** |  | | |  |  |  |  |  |  |
| *Lm*STAT1 | CL2896.Contig2_All | | | |  | 3231 | 788 | 0 | 73 |
| *Lm*STAT2 | CL2896.Contig1_All | | | |  | 3054 | 748 | 0 | 75 |
| **SOCS** |  | | |  |  |  |  |  |  |
| *Lm*SOCS1 | Unigene10563_All | | | |  | 1851 | 323 | 1.00E-62 | 73 |
| *Lm*SOCS2 | Unigene46_All | | |  |  | 3318 | 698 | 0 | 80 |
| *Lm*SOCS3 | Unigene18683_All | | | 1.3315 |  | 4224 | 517 | 8.00E-161 | 88 |
| *Lm*SOCS4 | CL2269.Contig2_All | | | 2.0358 |  | 3060 | 517 | 5.00E-161 | 88 |
| *Lm*SOCS5 | Unigene7482_All | | | 1.381 |  | 1610 | 274 | 1.00E-64 | 52 |
| *Lm*SOCS6 | Unigene29311_All | | | |  | 1341 | 234 | 2.00E-64 | 52 |
| *Lm*SOCS7 | CL2708.Contig2_All | | | |  | 3422 | 284 | 9.00E-154 | 92 |
| *Lm*SOCS8 | CL2708.Contig1_All | | | 4.6335 |  | 3272 | 284 | 9.00E-154 | 92 |
| *Lm*SOCS9 | Unigene8360_All | | | |  | 2437 | 224 | 4.00E-36 | 34 |
| *Lm*SOCS10 | Unigene2577_All | | | |  | 1539 | 239 | 2.00E-18 | 32 |
| *Lm*SOCS11 | Unigene19913_All | | | |  | 827 | 67 | 7.00E-08 | 34 |
| *Lm*SOCS12 | Unigene4819_All | | | 1.0648 |  | 3338 | 198 | 1.00E-44 | 33 |
| *Lm*SOCS13 | CL1824.Contig1_All | | | |  | 1901 | 568 | 2.00E-112 | 44 |
| *Lm*SOCS14 | Unigene18899_All | | | |  | 2361 | 457 | 0 | 36 |
| **PIAS** |  | | |  |  |  |  |  |  |
| *Lm*PIAS1 | CL3904.Contig3_All | | | -2.1921 |  | 2819 | 545 | 0 | 65 |
| *Lm*PIAS2 | CL3904.Contig2_All | | | |  | 3550 | 474 | 2.00E-155 | 63 |
| *Lm*PIAS3 | CL3904.Contig4_All | | | |  | 2774 | 467 | 1.00E-155 | 67 |
| *Lm*PIAS4 | CL3904.Contig1_All | | | |  | 3673 | 453 | 1.00E-153 | 63 |
| *Lm*PIAS5 | Unigene10598_All | | | |  | 4114 | 655 | 0 | 37 |
| *Lm*PIAS6 | CL3524.Contig2_All | | | 1.2862 |  | 4417 | 160 | 8.00E-25 | 71 |
| **Effectors** |  | | |  |  |  |  |  |  |
| **Prophenoloxidases** |  | | |  |  |  |  |  |  |
| *Lm*PPO1 | Unigene7093_All | | | -1.1669 |  | 2505 | 691 | 0 | 99 |
| *Lm*PPO2 | Unigene1020_All | | | |  | 2466 | 669 | 0 | 58 |
| *Lm*PPO3 | CL3897.Contig1_All | | | |  | 2504 | 659 | 0 | 57 |
| *Lm*PPO4 | Unigene7111_All | | | |  | 2544 | 663 | 0 | 58 |
| *Lm*PPO5 | CL3897.Contig2_All | | | |  | 1044 | 286 | 6.00E-98 | 60 |
| *Lm*PPO6 | Unigene3953_All | | | |  | 354 | 116 | 5.00E-38 | 65 |
| *Lm*PPO7 | Unigene35109_All | | | |  | 264 | 87 | 2.00E-39 | 80 |
| *Lm*PPO8 | Unigene22639_All | | | |  | 236 | 78 | 1.00E-25 | 67 |
| *Lm*PPO9 | Unigene34817_All | | | |  | 219 | 71 | 2.00E-28 | 83 |
| *Lm*PPO10 | Unigene25709_All | | | -3.1615 |  | 207 | 69 | 2.00E-25 | 84 |
| **lysozyme** |  | | |  |  |  |  |  |  |
| *Lm*Lys1 | CL3074.Contig3_All | | | -3.3784 |  | 647 | 139 | 4.00E-68 | 85 |
| *Lm*Lys2 | CL3074.Contig2_All | | | |  | 580 | 139 | 3.00E-68 | 85 |
| *Lm*Lys3 | CL3074.Contig1_All | | | |  | 620 | 139 | 4.00E-68 | 85 |
| *Lm*Lys4 | CL1703.Contig3_All | | | 3.7297 | 1.5442 | 2350 | 102 | 3.00E-35 | 37 |
| *Lm*Lys5 | CL1723.Contig4_All | | | |  | 2768 | 142 | 3.00E-34 | 51 |
| *Lm*Lys6 | CL1723.Contig3_All | | | |  | 2643 | 142 | 3.00E-34 | 51 |
| *Lm*Lys7 | Unigene19316_All | | | -2.1759 |  | 573 | 141 | 1.00E-25 | 44 |
| **AMP** |  | | |  |  |  |  |  |  |
| *Lm*Defensin | Unigene27068_All | | | |  | 246 | 62 | 5.00E-07 | 52 |
| *Lm*Diptericin | Unigene17444_All | | | |  | 459 | 93 | 1.00E-45 | 95 |
| **Nitric oxide synthase gene (NOS)** | | | | |  |  |  |  |  |
| *Lm*NOS1 | Unigene6853_All | | | | 1.1546 | 1019 | 298 | 2.00E-142 | 71 |
| *Lm*NOS2 | Unigene7425_All | | | | 1.2752 | 671 | 223 | 2.00E-103 | 70 |
| *Lm*NOS3 | Unigene34229_All | | | |  | 229 | 76 | 1.00E-24 | 68 |
| *Lm*NOS4 | Unigene26626_All | | | 1.4672 |  | 211 | 55 | 1.00E-16 | 76 |
| *Lm*NOS5 | Unigene16179_All | | | 1.5423 |  | 988 | 45 | 2.00E-11 | 58 |
| **NAPDH oxidase (NOX)** | |  |  | |  |  |  |  |  |
| *Lm*NOX1 | Unigene7015_All | | | |  | 1071 | 350 | 3.00E-134 | 86 |
| *Lm*NOX2 | Unigene9441_All | | | |  | 833 | 197 | 2.00E-113 | 32 |
| *Lm*NOX3 | Unigene2027_All | | | |  | 484 | 161 | 2.00E-87 | 41 |
| *Lm*NOX4 | Unigene20878_All | | | |  | 263 | 87 | 1.00E-16 | 89 |
| *Lm*NOX5 | Unigene19849_All | | | |  | 525 | 161 | 1.00E-29 | 30 |
| *Lm*NOX6 | Unigene3670_All | | | |  | 320 | 106 | 3.00E-53 | 36 |
| *Lm*NOX7 | Unigene22515_All | | | |  | 394 | 128 | 1.00E-60 | 87 |
| **Peroxidase (Pox)** |  | | |  |  |  |  |  |  |
| *Lm*POX1 | Unigene5499_All | | | |  | 2082 | 564 | 3.00E-116 | 41 |
| *Lm*POX2 | Unigene735_All | | | -1.4255 |  | 1866 | 563 | 9.00E-112 | 39 |
| *Lm*POX3 | CL780.Contig1_All | | | |  | 1923 | 433 | 4.00E-125 | 51 |
| *Lm*POX4 | CL780.Contig2_All | | | |  | 2356 | 437 | 5.00E-126 | 50 |
| *Lm*POX5 | Unigene6616_All | | | |  | 2522 | 381 | 2.00E-138 | 62 |
| *Lm*POX6 | CL941.Contig2_All | | | |  | 1667 | 525 | 1.00E-165 | 56 |
| *Lm*POX7 | Unigene2774_All | | | |  | 1361 | 319 | 5.00E-79 | 29 |
| *Lm*POX8 | CL1643.Contig1_All | | | |  | 1861 | 571 | 0 | 85 |
| *Lm*POX9 | Unigene2521_All | | | |  | 1189 | 391 | 1.00E-168 | 72 |
| *Lm*POX10 | Unigene15259_All | | | |  | 1429 | 397 | 1.00E-152 | 65 |
| *Lm*POX11 | Unigene26823_All | | | |  | 1438 | 428 | 1.00E-98 | 44 |
| *Lm*POX12 | Unigene5330_All | | | |  | 956 | 317 | 1.00E-64 | 42 |
| *Lm*POX13 | Unigene20446_All | | | 4.8499 |  | 1077 | 351 | 3.00E-74 | 43 |
| *Lm*POX14 | Unigene26706_All | | | |  | 215 | 71 | 3.00E-20 | 59 |
| *Lm*POX15 | Unigene12058_All | | | |  | 374 | 115 | 9.00E-23 | 45 |
| *Lm*POX16 | CL1643.Contig2_All | | | |  | 286 | 94 | 1.00E-44 | 93 |
| *Lm*POX17 | Unigene6001_All | | | |  | 451 | 147 | 1.00E-19 | 36 |
| *Lm*POX18 | Unigene21560_All | | | |  | 1471 | 187 | 2.00E-78 | 72 |
| *Lm*POX19 | Unigene15122_All | | | |  | 2653 | 158 | 2.00E-78 | 85 |
| *Lm*POX20 | Unigene2073_All | | | |  | 2133 | 118 | 9.00E-29 | 55 |
| *Lm*POX21 | Unigene9079_All | | | |  | 880 | 73 | 1.00E-13 | 40 |
| *Lm*POX22 | Unigene9356_All | | | |  | 380 | 126 | 1.00E-48 | 68 |
| *Lm*POX23 | Unigene15770_All | | | |  | 497 | 158 | 3.00E-49 | 59 |
| *Lm*POX24 | CL3965.Contig1_All | | | |  | 1533 | 346 | 4.00E-47 | 32 |
| *Lm*POX25 | Unigene15408_All | | | |  | 1021 | 286 | 7.00E-56 | 39 |
| *Lm*POX26 | CL3965.Contig3_All | | | |  | 1134 | 289 | 6.00E-40 | 32 |
| *Lm*POX27 | CL3965.Contig2_All | | | |  | 1088 | 289 | 6.00E-40 | 32 |
| *Lm*POX28 | Unigene21415_All | | | |  | 265 | 87 | 7.00E-15 | 49 |
| *Lm*POX29 | Unigene8248_All | | | |  | 443 | 146 | 9.00E-63 | 77 |
| *Lm*POX30 | Unigene21414_All | | | |  | 446 | 137 | 1.00E-25 | 49 |
| *Lm*POX31 | Unigene16545_All | | | |  | 437 | 85 | 2.00E-19 | 54 |
| *Lm*POX32 | Unigene21435_All | | | |  | 345 | 105 | 4.00E-30 | 57 |
| *Lm*POX33 | Unigene21434_All | | | |  | 211 | 56 | 3.00E-10 | 54 |
| *Lm*POX34 | Unigene21416_All | | | |  | 268 | 86 | 1.00E-13 | 42 |
| *Lm*POX35 | Unigene32016_All | | | |  | 224 | 71 | 2.00E-11 | 45 |
| *Lm*POX36 | Unigene20651_All | | | |  | 412 | 137 | 1.00E-55 | 74 |
| *Lm*POX37 | Unigene21843_All | | | |  | 206 | 65 | 2.00E-20 | 69 |
| **Superoxide Dismutases (SOD)** | | | |  |  |  |  |  |  |
| *Lm*SOD1 | Unigene19461_All | | | 1.5663 |  | 897 | 180 | 2.00E-53 | 56 |
| *Lm*SOD2 | Unigene14866_All | | | 1.2713 |  | 1669 | 170 | 2.00E-48 | 55 |
| *Lm*SOD3 | CL3068.Contig2_All | | | |  | 740 | 153 | 1.00E-58 | 70 |
| *Lm*SOD4 | Unigene14717_All | | | |  | 1705 | 154 | 1.00E-85 | 100 |
| *Lm*SOD5 | CL3068.Contig1_All | | | 5.0368 |  | 728 | 177 | 1.00E-54 | 60 |
| *Lm*SOD6 | Unigene7306_All | | | |  | 977 | 256 | 2.00E-83 | 60 |
| *Lm*SOD7 | Unigene15607_All | | | |  | 765 | 125 | 1.00E-37 | 54 |
| *Lm*SOD8 | Unigene19069_All | | | |  | 1111 | 211 | 1.00E-90 | 74 |
| *Lm*SOD9 | Unigene29766_All | | | |  | 620 | 192 | 2.00E-63 | 59 |
| *Lm*SOD10 | Unigene29758_All | | | |  | 787 | 191 | 5.00E-69 | 59 |
| *Lm*SOD11 | Unigene31988_All | | | |  | 537 | 172 | 9.00E-30 | 37 |
| *Lm*SOD12 | Unigene6499_All | | | |  | 1949 | 396 | 5.00E-30 | 29 |
| **Catalases** |  | | |  |  |  |  |  |  |
| *Lm*CAT1 | Unigene11123_All | | | |  | 1935 | 516 | 0 | 97 |
| *Lm*CAT2 | Unigene26933_All | | | |  | 1061 | 351 | 3.00E-122 | 60 |
| *Lm*CAT3 | Unigene29582_All | | | |  | 526 | 131 | 4.00E-22 | 40 |
| **Thioredoxin reductase** | | | |  |  |  |  |  |  |
| *Lm*TrxR1 | Unigene3066_All | | | |  | 870 | 195 | 6.00E-87 | 79 |
| *Lm*TrxR2 | Unigene6786_All | | | |  | 1540 | 211 | 8.00E-90 | 79 |
| *Lm*TrxR3 | Unigene14833_All | | | |  | 1881 | 242 | 7.00E-104 | 84 |
| *Lm*TrxR4 | CL299.Contig1_All | | | |  | 3361 | 486 | 0 | 73 |
| *Lm*TrxR5 | CL299.Contig2_All | | | |  | 2371 | 497 | 0 | 73 |
| *Lm*TrxR6 | CL299.Contig4_All | | | |  | 3436 | 511 | 0 | 73 |
| *Lm*TrxR7 | CL299.Contig3_All | | | |  | 2446 | 522 | 0 | 73 |
| *Lm*TrxR8 | Unigene532_All | | |  |  | 2370 | 584 | 0 | 60 |
| *Lm*TrxR9 | Unigene29900_All | | | |  | 1465 | 442 | 1.00E-128 | 49 |
| *Lm*TrxR10 | Unigene8451_All | | | |  | 6420 | 1981 | 0 | 23 |
| *Lm*TrxR11 | Unigene30976_All | | | |  | 382 | 126 | 2.00E-31 | 27 |
| *Lm*TrxR12 | CL4483.Contig2_All | | | |  | 511 | 98 | 1.00E-21 | 47 |
| *Lm*TrxR13 | CL4483.Contig1_All | | | |  | 509 | 98 | 1.00E-21 | 47 |
| *Lm*TrxR14 | CL841.Contig4_All | | | |  | 922 | 92 | 1.00E-31 | 43 |
| *Lm*TrxR15 | CL841.Contig3_All | | | |  | 1013 | 92 | 1.00E-31 | 43 |
| *Lm*TrxR16 | CL841.Contig2_All | | | |  | 991 | 92 | 1.00E-31 | 43 |
| *Lm*TrxR17 | CL841.Contig1_All | | | |  | 1013 | 92 | 1.00E-31 | 43 |
| *Lm*TrxR18 | Unigene5157_All | | | |  | 1082 | 132 | 1.00E-50 | 34 |
| *Lm*TrxR19 | Unigene30321_All | | | |  | 289 | 95 | 1.00E-28 | 31 |
| **Peroxiredoxin** |  | | |  |  |  |  |  |  |
| *Lm*Prx1 | Unigene3066_All | | | |  | 870 | 195 | 6.00E-87 | 79 |
| *Lm*Prx2 | Unigene6786_All | | | |  | 1540 | 211 | 8.00E-90 | 79 |
| *Lm*Prx3 | Unigene14833_All | | | |  | 1881 | 242 | 7.00E-104 | 84 |
| *Lm*Prx4 | Unigene29536_All | | | |  | 716 | 200 | 1.00E-114 | 60 |
| *Lm*Prx5 | Unigene11064_All | | | |  | 1612 | 219 | 1.00E-90 | 71 |
| *Lm*Prx6 | CL1371.Contig2_All | | | |  | 787 | 220 | 9.00E-96 | 85 |
| *Lm*Prx7 | Unigene17562_All | | | |  | 1222 | 172 | 6.00E-67 | 74 |
| *Lm*Prx8 | Unigene30726_All | | | |  | 617 | 143 | 1.00E-27 | 48 |
| *Lm*Prx9 | CL1371.Contig3_All | | | -1.4634 |  | 237 | 64 | 2.00E-31 | 78 |
| *Lm*Prx10 | CL1371.Contig1_All | | | |  | 227 | 58 | 8.00E-27 | 81 |
| *Lm*Prx11 | Unigene11383_All | | | |  | 331 | 110 | 9.00E-23 | 64 |
| **Other immune molecules** | | | |  |  |  |  |  |  |
| **Caspase homology** |  | | |  |  |  |  |  |  |
| *Lm*Caspase1 | CL2922.Contig2_All | | | 3.3031 |  | 1181 | 274 | 8.00E-81 | 55 |
| *Lm*Caspase2 | CL2922.Contig1_All | | | 11.1308 |  | 1349 | 274 | 9.00E-81 | 55 |
| *Lm*Caspase3 | CL2917.Contig2_All | | | |  | 1971 | 271 | 8.00E-100 | 65 |
| *Lm*Caspase4 | CL2917.Contig1_All | | | |  | 1952 | 271 | 7.00E-100 | 65 |
| *Lm*Caspase5 | Unigene17158_All | | | |  | 1949 | 166 | 1.00E-36 | 46 |
| *Lm*Caspase6 | Unigene3368_All | | | -6.7444 |  | 1184 | 278 | 6.00E-42 | 38 |
| *Lm*Caspase7 | CL1432.Contig3_All | | | -1.5629 |  | 2116 | 428 | 3.00E-56 | 33 |
| *Lm*Caspase8 | CL1432.Contig2_All | | | -2.0303 |  | 1923 | 384 | 2.00E-53 | 30 |
| *Lm*Caspase9 | CL1432.Contig1_All | | | |  | 1928 | 428 | 3.00E-56 | 33 |
| *Lm*Caspase10 | Unigene146_All | | |  |  | 3906 | 532 | 2.00E-71 | 34 |
| *Lm*Caspase11 | Unigene37454_All | | | |  | 365 | 101 | 1.00E-20 | 50 |
| *Lm*Caspase12 | Unigene35981_All | | | |  | 229 | 74 | 1.00E-12 | 42 |
| *Lm*Caspase13 | Unigene19937_All | | | |  | 624 | 132 | 3.00E-15 | 34 |
| *Lm*Caspase14 | Unigene12677_All | | | |  | 1432 | 124 | 5.00E-14 | 35 |
| *Lm*Caspase15 | Unigene35593_All | | | |  | 530 | 68 | 2.00E-09 | 43 |
| *Lm*Caspase16 | CL1123.Contig2_All | | | |  | 3467 | 643 | 6.00E-81 | 32 |
| *Lm*Caspase17 | Unigene28482_All | | | |  | 342 | 73 | 1.00E-08 | 44 |
| *Lm*Caspase18 | CL1432.Contig2_All | | | -2.0303 |  | 1923 | 384 | 2.00E-53 | 30 |
| **Dicer** |  | | |  |  |  |  |  |  |
| *Lm*Dicer1 | CL4452.Contig2_All | | | |  | 6144 | 1620 | 0 | 42 |
| *Lm*Dicer2 | CL4621.Contig2_All | | | |  | 5686 | 357 | 6.00E-173 | 82 |
| *Lm*Dicer3 | Unigene37366_All | | | |  | 257 | 46 | 6.00E-19 | 100 |
| *Lm*Dicer4 | Unigene37908_All | | | |  | 225 | 74 | 5.00E-36 | 100 |
| **Argonaute** |  | | |  |  |  |  |  |  |
| LmAGO2-1 | CL2119.Contig1_All | | | |  | 3005 | 971 | 0 | 44 |
| LmAGO2-2 | CL2119.Contig2_All | | | |  | 3151 | 980 | 0 | 44 |
| LmAGO2-3 | CL2127.Contig1_All | | | -1.6314 |  | 696 | 219 | 7.00E-78 | 67 |
| LmAGO2-4 | CL2127.Contig2_All | | | -1.2996 |  | 1119 | 219 | 4.00E-80 | 65 |
| LmAGO2-5 | CL2741.Contig2_All | | | |  | 1457 | 358 | 3.00E-29 | 27 |
| LmAGO2-6 | CL3524.Contig1_All | | | |  | 3206 | 184 | 2.00E-10 | 34 |
| LmAGO2-7 | Unigene17619_All | | | |  | 1740 | 254 | 1.00E-54 | 48 |
| LmAGO2-8 | Unigene635_All | | |  |  | 1945 | 532 | 0 | 88 |
| LmAGO2-9 | Unigene77_All | | |  |  | 3268 | 873 | 0 | 96 |
