# Supplementary material for: Comparative transcriptomic analysis of immune responses of the migratory locust, Locusta migratoria, to challenge by the fungal insect pathogen, Metarhizium acridum
Source: BMC Genomics. 2015 Oct 26;16:867. doi: 10.1186/s12864-015-2089-9 (PMC4624584; doi:10.1186/s12864-015-2089-9)
Supplement: Additional file 2: Table S2. — qRT-PCR primers. (DOCX 16 kb) [file 12864_2015_2089_MOESM2_ESM.docx]

**Additional file 2: Table S2** qRT-PCR primers

| Genes | Forward (5’-3’) | Reverse (5’-3’) |
| --- | --- | --- |
| LmPGRP1 | TCGGAGACTTCACAGATA | TGATAGAGAGCCAATCCA |
| LmPGRP2 | TTATGAAGGTCGTGGATG | TCTTCTGGTGGAGTAACA |
| LmCTL8 | CAGAAGGAGAATACATCACT | TTAGCCTCTTGGTCAATAC |
| LmCTL11 | CGACTACTGAGTACAAGAC | CTCCTTGGAGTTGATGAC |
| LmCTL14 | CGTCTCAATACAACTCTATATG | ATTGCCAAGTACAATCTCT |
| LmSCRB11 | ACTTCTACTTCTGGAACTG | ACGTGATGTTGATCTTCT |
| LmSCRB12 | GGCGGTATTAACAGAAGA | GTCACGATTCCTCCAATA |
| LmDSCAM9 | GAGAGTGAACCAGAAGAG | CCAAGACCAGCAATAAGA |
| LmDSCAM14 | GAAGACCTATCCAGATGAC | ATATGTATGTTCCAGAATGTTC |
| LmDSCAM18 | GTTGAAGTGGAAGGAGTT | AGGGTATATGTTTCTGAAGTT |
| LmDSCAM69 | GAACCAGTAGCATCTCAG | GCATTATCACAGCGACTA |
| LmDSCAM76 | TCAGGTGCTTGCTTATAC | ACAGTCTTATGCCAATGAA |
| LmSRPN8 | CTGGAGTAGAGGAAGTAGA | GAGTTAAGCCATTCAATATACC |
| LmSRPN12 | CTCGCTTAGTTCTTGTCA | GCTCTTCTGCTTCTGTATA |
| LmSRPN13 | TCTATACTTCATCATCTCAATCT | TTAGGCAATATCACCATCAT |
| LmSRPN23 | CGCCATATACTTCAACGG | GGTAACCTCAAGGACTTC |
| LmSRPN27 | TGGTGTTGGTTAATGCTAT | ACTTGACTATTCAGATCACTTA |
| LmSRPN30 | AAAGAACTGACGAGTGAG | GCTTCAAAGAAAGACTTATGT |
| LmSRPN33 | AAACATCTCATTTACTCTCTAAG | AATATCACCTTCTGCCATAA |
| LmTLR3 | TCAAAGAGCTGCACATTC | CAGGAGATCGTCAGGAAG |
| LmTLR14 | GTATCGCATCTGCCTTTA | TCTTTGAGAGGACGACTA |
| *Lm*Tollip1 | GTGTGACAAGCATATACCT | AAGTTAATCATTCCTTCCATTC |
| LmPellino3 | ACTCTGACTCTACCACAA | ATGTTGCGTAAGTTCTCA |
| LmPellino4 | ACTCTGACTCTACCACAA | ATGTTGCGTAAGTTCTCA |
| LmPellino6 | ACTCTGACTCTACCACAA | ATGTTGCGTAAGTTCTCA |
| LmPellino7 | ACTCTGACTCTACCACAA | ATGTTGCGTAAGTTCTCA |
| LmPellino8 | ACTCTGACTCTACCACAA | ATGTTGCGTAAGTTCTCA |
| LmPellino11 | ATATTATGGAGATGGTGTGAA | CTGACCTGACTGAAATAGAT |
| *Lm*IMD1 | GAAGCACAGTTCCAGTTA | GCCATCCAAGGTATAATCT |
| *Lm*Domeless2 | GACCTCTATGAACGGAAA | GTGACTGGTTCTGTGTAA |
| LmSOCS3 | TGAATCTATCTCCAGGAATAC | ACCCTCTAACAATCTCTCT |
| LmSOCS4 | TGAATCTATCTCCAGGAATAC | ACCCTCTAACAATCTCTCT |
| LmSOCS5 | AGGTCATATACAGCACAAC | ACACCATTGGATATAGAACTT |
| LmSOCS8 | GTAGCAACGATCAGTCAT | AGAGTGTACCATCATCCA |
| LmSOCS12 | CTGACTCGTCCACTCTAT | GTAAGGATATTCGCTGAGAT |
| LmPIAS1 | GTGACTCTGATGATGATACA | TACTACTAACACAGGATGGA |
| LmPIAS6 | TGAAGAGTATGGTTCTAAGC | CATTGTTACAGGTGTTGAA |
| *Lm*PPO1 | AGACAACGAAGGCTTATC | CTGTATCTGCTGCTTGAT |
| *Lm*PPO10 | CACCATTTGTAACCAGAATT | CGTAACAACATTGGAAGTC |
| LmLys1 | CCAGATCAACAGCAAGTA | TGTTCAGCAGATCTTCAC |
| LmLys7 | GAGAGTGAGAGCTACCTA | AGTCTCCACACCAGTATT |
| LmNOS4 | TTACTGGAAGTCTTGGAAG | AGAAGATGAAATGCTGTAGA |
| LmNOS5 | GCAACAGACCTGAAATTG | CCATTGGACGTGTATTCT |
| LmPOX2 | AGGATGATAATCTGGATGAG | GCCAATAAGTTCCCGATA |
| LmPOX13 | ACAGGTTCTTCTATGATTACA | GTTACTGGATTCGTGTCA |
| LmSOD1 | AATCCTGACAAGGTGAAC | TCTGAGTGAACAACAACA |
| LmSOD2 | AATCCTGACAAGGTGAAC | TCTGAGTGAACAACAACA |
| LmSOD5 | GATTGGTGAGGTAGTGAAT | TAATGATGTTAGGGATGGAATA |
| LmPrx9 | TACAAGTGGAAGGGAAAC | TTCTGGAATTCTGGGTTG |
| LmCaspase1 | CATATTCAGTGGACTATCTCT | TTCTTGAGCGAACTAACTT |
| LmCaspase2 | CATATTCAGTGGACTATCTCT | TTCTTGAGCGAACTAACTT |
| LmCaspase6 | GAAGAAGGCACTCCATAC | CAAGGTTGAAGTGTAGCA |
| LmCaspase7 | ATCTCCACCTCCACATAA | TGAACACCTGACATATCG |
| LmCaspase8 | ATCTCCACCTCCACATAA | TGAACACCTGACATATCG |
| LmAGO2-3 | ATAGAGATGGAGTGAGTGA | AATGTAATGGCAGGTTCA |
| LmAGO2-4 | ATAGAGATGGAGTGAGTGA | AATGTAATGGCAGGTTCA |
| LmNOS1 | CCTTCTCCTGCTTTGTTA | ACCATCTTGTGTTCTGTAG |
| LmNOS2 | GATGTGAGTTACCTTACCA | AGTATGTGTCTCCTTCAAC |
